# Dynamic Changes of the Anthocyanin Biosynthesis Mechanism During the Development of Heading Chinese Cabbage (*Brassica rapa* L.) and *Arabidopsis* Under the Control of *BrMYB2*

**DOI:** 10.3389/fpls.2020.593766

**Published:** 2020-12-23

**Authors:** Qiong He, Qianqian Lu, Yuting He, Yaxiu Wang, Ninan Zhang, Wenbin Zhao, Lugang Zhang

**Affiliations:** ^1^State Key Laboratory of Crop Stress Biology for Arid Areas, College of Horticulture, Northwest A&F University, Yangling, China; ^2^College of Life Sciences, Northwest A&F University, Yangling, China; ^3^State Key Laboratory of Vegetable Germplasm Innovation, Tianjin, China

**Keywords:** anthocyanin, *Arabidopsis*, *Brassica rapa*, color, development, gene expression, qRT-PCR

## Abstract

Chinese cabbage is an important vegetable mainly planted in Asian countries, and mining the molecular mechanism responsible for purple coloration in *Brassica* crops is fast becoming a research hotspot. In particular, the anthocyanin accumulation characteristic of purple heading Chinese cabbage, along with the plant’s growth and head developing, is still largely unknown. To elucidate the dynamic anthocyanin biosynthesis mechanism of Chinese cabbage during its development processes, here we investigated the expression profiles of 86 anthocyanin biosynthesis genes and corresponding anthocyanin accumulation characteristics of plants as they grew and their heads developed, between purple heading Chinese cabbage 11S91 and its breeding parents. Anthocyanin accumulation of 11S91 increased from the early head formation period onward, whereas the purple trait donor 95T2-5 constantly accumulated anthocyanin throughout its whole plant development. Increasing expression levels of *BrMYB2* and *BrTT8* together with the downregulation of *BrMYBL2.1*, *BrMYBL2.2*, and *BrLBD39.1* occurred in both 11S91 and 95T2-5 plants during their growth, accompanied by the significantly continuous upregulation of a phenylpropanoid metabolic gene, *BrPAL3.1*; a series of early biosynthesis genes, such as *BrCHS*s, *BrCHI*s, *BrF3H*s, and *BrF3’H*; as well as some key late biosynthesis genes, such as *BrDFR1*, *BrANS1*, *BrUF3GT2*, *BrUF5GT*, *Br5MAT*, and *Brp-Cout*; in addition to the transport genes *BrGST1* and *BrGST2*. Dynamic expression profiles of these upregulated genes correlated well with the total anthocyanin contents during the processes of plant growth and leaf head development, and results supported by similar evidence for structural genes were also found in the *BrMYB2* transgenic *Arabidopsis*. After intersubspecific hybridization breeding, the purple interior heading leaves of 11S91 inherited the partial purple phenotypes from 95T2-5 while the phenotypes of seedlings and heads were mainly acquired from white 94S17; comparatively in expression patterns of investigated anthocyanin biosynthesis genes, cotyledons of 11S91 might inherit the majority of genetic information from the white type parent, whereas the growth seedlings and developing heading tissues of 11S91 featured expression patterns of these genes more similar to 95T2-5. This comprehensive set of results provides new evidence for a better understanding of the anthocyanin biosynthesis mechanism and future breeding of new purple *Brassica* vegetables.

## Introduction

Color is a fundamental property of horticulture plants. Apart from providing ornamental value, gorgeous and eye-catching colors enable these plants to attract animal pollinators required for sexual reproduction and seed dispersal (Iwashina, 2015). Anthocyanins are the most important pigments in the flavonoid family, as they not only contribute to the wide range of light pink to deep purple colors but also furnish plants with potent radical scavenging capacities to protect against biotic and abiotic stresses (Iwashina, 2015; Zhai et al., 2019). Moreover, edible anthocyanins are well known for improving humans’ immune system responses and reducing their chronic disease risks; and furthermore, a high dose of anthocyanins is non-toxic and able to reduce the teratogenic and mutagenic incidence (Wiczkowski et al., 2014; Joo et al., 2018). Hence, anthocyanins are widely used in the food industry, and anthocyanin-rich foods or products are becoming more sought after and more prevalent because of their health benefits and ornamental value to humans (Xu Z. S. et al., 2020).

The anthocyanin biosynthesis pathway has been well investigated in many horticultural species: ornamental plants include roses and petunias; fruit plants include apples, strawberries, cherries, blueberries, pears, peaches, and grapes; vegetable plants include cabbages, eggplants, carrots, radishes, and onions (Naing and Kim, 2018; Peng et al., 2019; Wang et al., 2020). This biosynthesis pathway involves a series of reactions catalyzed by diverse enzymes, occurring in three main steps. The first phenylpropanoid metabolic pathway involves phenylalanine ammonia lyase (PAL), cinnamate 4-hydroxylase (C4H), and 4-coumarate: CoA ligase (4CL). Participating in the second early biosynthesis pathway are chalcone synthase (CHS), chalcone isomerase (CHI), flavanone-3-hydroxylase (F3H), flavanone 3′-hydroxylase (F3′H) or flavanone 3′5′-hydroxylase (F3′5′H), and side-branching enzymes for flavonols, isoflavones, and flavones production such as flavonol synthase (FLS), isoflavone synthase (IFS), and flavone synthase (FNS). The third late biosynthesis pathway is the process of synthesis and modification of anthocyanins, carried out by dihydroflavonol 4-reductase (DFR), anthocyanin synthase (ANS), UDP-glucosyltransferase (UGT), and acyltransferase (AT) (Liu et al., 2018; Sun et al., 2020). Meanwhile, dynamic competition between FLS and DFR will lead to either flavonol synthesis or anthocyanin accumulation in subsequent processes (Li et al., 2020), and processes catalyzed by anthocyanin reductase (ANR) and leucoanthocyanidin reductase (LAR) will generate the accumulation of proanthocyanidins (Lepiniec et al., 2015).

Apart from the above structural enzymes central to the process of anthocyanin synthesis, many regulators reportedly participate in how the anthocyanin biosynthesis pathway is governed and activated. For example, functional R2R3-MYB transcription factors, such as MYB11, MYB12, and MYB111, are generally considered responsible for the direct induction of early biosynthesis genes (EBGs), namely *CHS*, *CHI*, *F3H*, *F3′H*, and *FLS* (Guo et al., 2014). Yet the late biosynthesis genes (LBGs) such as *DFR*, *ANS*, *UGT*s, and *AT*s, are usually activated by a MYB-bHLH-WD40 ternary complex—MBW, usually formed by an R2R3-MYB factor, a basic-helix-loop-helix factor (bHLH), and a WD40-repeat factor—and the *F3’H* acts as an intermediate in the anthocyanin synthesis process since it can be activated by either MYBs functioning in the EBGs or by regulators affecting in the LBGs (Guo et al., 2014; Xu Z. S. et al., 2020). Several reports also indicate that the MYBs or MBW complex has the redundant ability to activate members of the phenylpropanoid biosynthesis pathway (Choo et al., 2013; Liu et al., 2015; Alan et al., 2017). Nonetheless, the MYBs will usually show some functional redundancy with more than one class of products during the biosynthesis process: for example, AtMYB4 regulates the production of monolignols and phenolic acids while AtMYB12 activates both flavonol and phenolic acid biosynthesis in *Arabidopsis*, whereas AtPAP1 has the ability to accumulate monolignols, anthocyanins, proanthocyanidins, flavonols, and phenolic acids; VvMYB5a is responsible for the accumulation of anthocyanins, proanthocyanidins, and flavonols, while VvMYB5b mainly controls the production of anthocyanins and proanthocyanidins in grapevine; in potato, both StAN1 and StMTF1 are able to activate the biosynthesis of anthocyanins, flavonols, and phenolic acids (Liu et al., 2015). Apart from these positive MYBs, bHLHs, and WD40s are also crucial for the formation of the MBW complex and activation of the anthocyanin synthesis pathway (Nuraini et al., 2020). The first clue inspiring the formation of the canonical MBW complex in the regulation of flavonoid synthesis can date back to the discovery of transposable elements in the 1950s (Alan et al., 2017). Numerous studies have shown that TTG1 (WD40), GL3/EGL3/TT8 (bHLH), and PAP1/PAP2/MYB113/MYB114 (MYB) are key components of potential MBW complexes that activate anthocyanin biosynthesis (Xie et al., 2012; Xu Z. S. et al., 2020). The bHLHs often function as countershafts in the interaction with MYBs and WD40s, with WD40 playing a key role in promoting the coaction of bHLHs and MYBs; other work also revealed the MYBs to primarily regulate flavonoid synthesis pathway genes, while bHLHs and WD40s often bind to sites upstream of these MYBs (Zhao et al., 2013; Guo et al., 2014; Liu et al., 2015; Alan et al., 2017).

In addition to these positive regulators, existed negative regulators could also influence the regulation of anthocyanin biosynthesis, in that they suppress the transcription and expression of target genes by binding to specific DNA sequences of their promoter regions, thereby indirectly modulating protein–protein interactions or genes’ co-expression in the flavonoid synthesis pathway (Han et al., 2020). For instance, the R3-MYBs ‘MYBL2’ and ‘CPC’ (CAPRICE), together with the lateral organ boundary domain (LBD) factors ‘LBD37,’ ‘LBD38,’ and ‘LBD39,’ are several well-studied negative regulators of anthocyanin biosynthesis in *Arabidopsis*, which repress anthocyanin biosynthesis either by suppressing the anthocyanin biosynthesis genes (ABGs) or by directly inhibiting the formation of MBW complexes (Zhu et al., 2009; Dubos et al., 2010; Matsui et al., 2010; Chen et al., 2019). A notable example is AtMYBL2, occurs in seeds or vegetative tissues, inhibits the upregulation of *AtTT8*, *AtPAP1*, and *AtPAP2*; moreover, AtTT8 is able to positively upregulate the *AtMYBL2* gene (Dubos et al., 2010; Matsui et al., 2010). Another classic case of interaction between negative repressors and positive regulators with the spatial patterning considered that these interactions happen in a local, autocatalytic feedback loop and a long-range inhibitory feedback loop: an R2R3-MYB activator and an R3-MYB repressor constitute a double “activator and suppressor” components reaction-diffusion system in monkey flower, and this system dynamically interacts with MlANbHLH1 to regulate the formation of dispersed anthocyanin spots in its petals (Ding et al., 2020). In this process, activators promote color generation and repressors restrain color formation, and they meet and react, and thereby producing pigments diffusion (Ding et al., 2020). It follows that these regulators or complexes are capable of binding to the promoters of structural genes or regulatory genes, thereby controlling their upregulation and downregulation, and the regulation network of flavonoid biosynthesis pathway is operated by a feedback–regulation mechanism in which positive and negative regulators participate and interact. These dynamic processes might corporately govern the accumulation and reduction of anthocyanins, proanthocyanidins, or flavonols. For example, a heterodimer of the NAC transcription factor BL, together with the PpNAC1, activates the transcription of *PpMYB10.1* and results in the anthocyanin pigmentation in blood-fleshed peach; however, another SQUAMOSA promoter-binding protein-like transcription factor, PpSPL1, represses the *BL-PpNAC1* heterodimer, thereby inhibiting the upregulation of *PpMYB10.1* in the peach fruit development (Zhou et al., 2015). Collectively, these described findings emphasize that the anthocyanin biosynthesis pathway is an extremely sophisticated metabolism pathway mediated by both activators and repressors during plants’ development.

**FIGURE 1:**
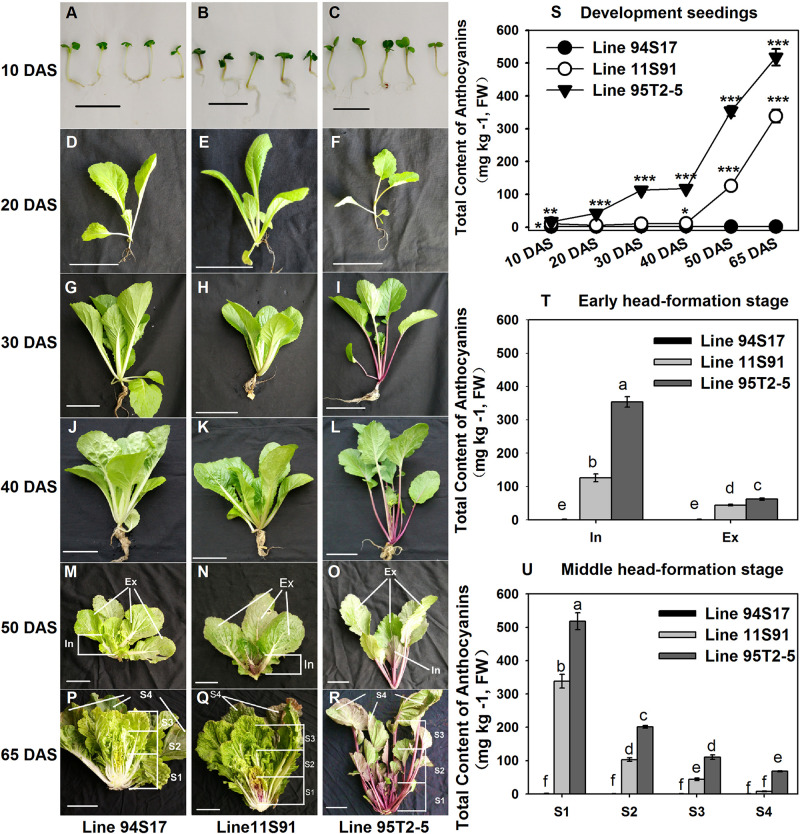
Phenotypes and total anthocyanin content of different development tissues of three Chinese cabbages. **(A,R)** Images in each vertical sample column (left to right) were collected from 94S17, 11S91, and 95T2-5. **(A–C)** 10 DAS (Day after sowing) seedlings; **(D–F)** 20 DAS seedlings; **(G–I)** 30 DAS seedlings; **(J–L)** 40 DAS seedlings. **(M–O)** 50 DAS plants at the early head-formation stage; the head of 11S91 was divided into two parts: In, the interior heading leaves with deep purple color; Ex, the external functional leaves. **(P–R)** 65 DAS plants at the middle head-formation stage; the head of 11S91 was divided into four parts: S1, interior heading leaves with a deep-purple color; S2, inner heading leaves with a light-purple color; S3, exterior heading leaves; S4, outer functional leaves. The leaf size and positions of 94S17 and 95T2-5 samples at both stages were collected in the same as 11S91. **(S)** Total anthocyanin content of the development samples, for which the ‘*’, ‘**’, and ‘***’ above each symbol indicate significant, highly significant, and extremely significant differences at *p* < 0.05, *p* < 0.01, and *p* < 0.001, respectively. **(T,U)** Total anthocyanin content of different heading samples, for which different letters above each column are significantly different at *p* < 0.05 according to Duncan’s test. The scale bar is 2 cm in **(A–C)** and 10 cm in **(D–R)**. The values shown are means ± SDs (*n* = 3).

Chinese cabbage (*Brassica rapa* L. ssp. *pekinensis*), an important member of the *Brassicaceae* family, is widely planted in Asian countries, being a meaningful native species of China, having white, yellow, orange or green heads. Yet the anthocyanin-rich Chinese cabbage type is actually rare due to the absence of novel natural mutants. Thus, purple heading Chinese cabbage is currently created via hybridization with other purple varieties and species in the *Brassicaceae* family, and the investigation of molecular mechanism of red or purple *Brassica* crops is emerging as a research hotspot. For example, a purple head Chinese cabbage that featured the deep-purple head and had purple genes on chromosome A02 was produced by the interspecific hybridization between a heading Chinese cabbage (2n = AA = 20) and a red-leaf mustard plant (*B. juncea*, 2n = AABB = 36); however, the purple-leaf phenotype is a qualitative trait and its inheritance character is unstable (Zhang et al., 2016). In another classic creation of reddish-purple Chinese cabbage, an interspecific crossing was made between a green Chinese cabbage (2n = AA = 20) and a red cabbage (*B. oleracea* L. var. *capitata* f. *rubra*, 2n = CC = 18), then combined with a colchicine treatment to form allotetraploids and a recurrent backcrossing, to acquire the red aneuploid individuals of Chinese cabbage (Lee et al., 2018). The new reddish-purple Chinese cabbage appeared as a red head phenotype with introduced dominant genes and a retarded growth character, whose transcriptome analysis showed the LBGs *BrDFR*, *BrLDOX*, *BrUF3GT*, *BrUGT75c1-1*, *Br5MAT*, *BrAT-1*, *BrAT-2*, *BrTT19-1*, and *BrTT19-2* and the regulatory MYB genes *BrMYB90*, *BrMYB75*, and *BrMYBL2-1* were highly expressed in it (Lee et al., 2018; Rameneni et al., 2020). Unlike those distant hybridization forms of breeding, we have created a novel, purple heading Chinese cabbage based on the inspiration of intersubspecific hybridization, for which a white heading Chinese cabbage (94S17, 2n = AA = 20) and a purple flowering Chinese cabbage (95T2-5, 2n = AA = 20) are used in the hybridization (He et al., 2016; Wu et al., 2016). Of course, our purple heading Chinese cabbage also has the special purple head features—the purple inner heading leaves, green outer heading leaves, normal growth, and stable genetic characters—but a different genetic background from previous reports of it created via the interspecific hybridization. Although, the key regulatory gene *BrMYB2* (Bra004162, located on chromosome A07) controlling the purple head trait of Chinese cabbage was verified recently (He et al., 2016, 2020b; Wu et al., 2016), we find that its leaves accumulated the purple coloring with spatial and temporal specificities during the processes of plant growth and head development (Figure 1). Despite much recent progresses, the biochemical and molecular basis for these changes remain elusive. Hence, we own an excellent opportunity to investigate the anthocyanin biosynthesis mechanism in the novel mutant during these developing processes.

**FIGURE 2:**
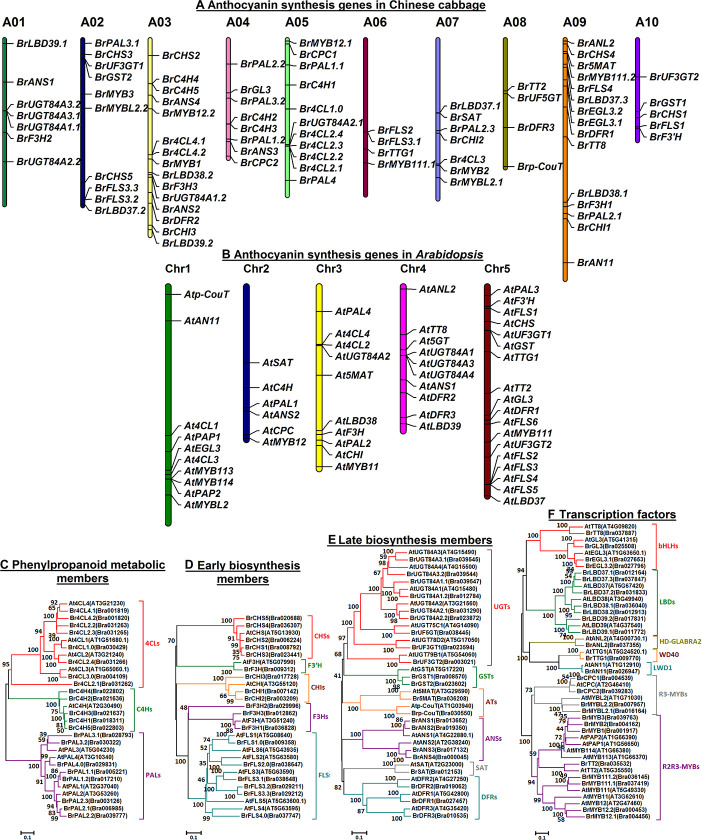
Chromosome distributions and phylogenetic trees of members involved in anthocyanin biosynthesis in Chinese cabbage and *Arabidopsis*. **(A,B)** Chromosome distributions of ABGs in Chinese cabbage **(A)** and *Arabidopsis* **(B)**; **(C–F)** the neighbor-joining phylogenetic trees for phenylpropanoid metabolic members **(C)**, early biosynthesis members **(D)**, late biosynthesis members **(E)**, and transcriptional factors **(F)**. Node numbers represent the bootstrap values from 1000 trials containing each clade, and the lengths of the branch lines indicate the extent of divergence. The chromosomal information and protein sequences were collected from the BRAD database.

Furthermore, the relevant ABGs usually have just one copy in *Arabidopsis*, whereas multiple ABG copies of *Brassica* crops are generated after whole genome duplication yet retain synteny with their orthologs from *Arabidopsis* (Guo et al., 2014). A total of 86 ABGs corresponding to ABG homologs of *Arabidopsis* were identified in heading Chinese cabbage and distributed in 10 chromosomes (Figure 2), and most LBGs and positive regulatory genes occur in fewer than three copies, though more phenylpropanoid metabolic pathway genes (PMPGs), EBGs and negative regulatory genes are generated (Guo et al., 2014; He et al., 2020a). In addition, members clustered in a clade with closer branch distances usually showed high homology and functional similarity (Figures 2C–F), but they might also show functional differences and expression specificity. For example, At4CL1, At4CL2, and At4CL4 are more closely to each other than At4CL3 (Figure 2C), and they mainly participated in the lignin biosynthesis in *Arabidopsis*; however, *At4CL1* and *At4CL2* are expressed in lignifying cells, *At4CL3* is expressed in a broad range of cell types and plays a distinct role in flavonoid metabolism, and *At4CL1* also showed similar function in this pathway (Li et al., 2015). Additionally, four *ANS*s and 12 *DFR*s of *B. rapa* were investigated under cold and freezing conditions: *BrANS2*, *BrDFR1*, *BrDFR3*, *BrDFR5*, *BrDFR6*, and *BrDFR10* genes all responded to cold and freezing stress treatments, whereas *BrANS1*, *BrANS3*, *BrDFR2*, *BrDFR4*, *BrDFR8*, and *BrDFR*9 only highly responded to cold stress in the purple-pigmented *B. rapa* plants (Ahmed et al., 2014, 2015). In the reddish purple head Chinese cabbage, results indicate that *BrPAL*, *BrPAL2*, *BrPAL4*, *BrC4H*, *Br4CL2*, *BrCHS*, *BrCHI*, *BrCHI1*, *BrF3H*, and *BrF3′H-1* may be involved in the early phase of anthocyanin biosynthesis; however, LBGs *BrDFR*, *BrLDOX*, *BrUF3GT*, *BrUGT75c1-1*, *Br5MAT*, *BrAT-1*, *BrAT-2*, *BrTT19-1*, and *BrTT19-2* and the regulatory MYB genes *BrMYB90*, *BrMYB75*, and *BrMYBL2-1* might play important roles in the anthocyanin biosynthesis in their purple plants (Rameneni et al., 2020). Hence, that evidence raises the pressing questions of whether all ABGs are involved in the purple trait formation and how do they operate during the development of the purple heading Chinese cabbage. In this study, dynamic changes of anthocyanin accumulation characteristics during plant development of three Chinese cabbage color types were determined, and the expression patterns of their corresponding ABGs in the anthocyanin biosynthesis pathway were also analyzed. In addition, related investigations and comparisons were also conducted using *BrMYB2*-transgenic *Arabidopsis*. The mechanism of purple color formation investigated here at both its physiological and molecular levels will supply a theoretical basis for advancing purple trait breeding efforts in the *Brassicaceae* family.

## Materials and Methods

### Plant Materials and Growth Conditions

The inbred line 94S17 with white leaf heads, the flowering Chinese cabbage line 95T2-5 with a deep-purple appearance, and the Chinese cabbage line 11S91 with stable inheritance of purple leaf heads were used (Figure 1). Notably, Chinese cabbage 11S91 was bred from a selected single plant of an F_1_ generation hybridized between 94S17 and 95T2-5 with continuous self-crossing for 10 generations.

To better understand the expression patterns of ABGs and related anthocyanin synthesis metabolisms, samples of the three lines were collected throughout their vegetative growth periods. Briefly, their seedlings were collected at four time-points: 10 DAS (days after sowing; the cotyledon stage, Figures 1A–C), 20 DAS (Figures 1D–F), 30 DAS (Figures 1G–I), and 40 DAS (Figures 1J–L). During the leaf head development, we collected samples at the early head-formation stage (ca. 50 DAS) and middle head-formation stage (ca. 65 DAS); the 50 DAS plants of 11S91 were then divided into two parts, namely interior purple leaves and external functional leaves. The 65 DAS plants of 11S91 were divided into four parts, spanning interior purple heading leaves to external functional leaves: S1, interior heading leaves with a deep-purple color; S2, inner heading leaves with a light-purple color; S3, exterior heading leaves; S4, outer functional leaves. The leaf size and positions of 94S17 and 95T2-5 samples at these two stages were collected in the same as done for 11S91. All the plants were grown outdoors, in autumn, in Yangling, Shaanxi Province (China).

To better understand the functioning of the key regulatory gene *BrMYB2* and related expression similarities and differences of ABGs, the *BrMYB2*-transgenic *Arabidopsis* lines were acquired from the authors of recently published study, and the growth conditions of these lines and wild type (WT) *Arabidopsis* Columbia were performed as previously described (He et al., 2020b). Briefly, we cloned the coding sequence of *BrMYB2* gene and transferred it into a pVBG2307 binary vector, and then transferred it into *Agrobacterium tumefaciens* strain GV3101 using the freeze-thaw method and transformed into WT *Arabidopsis* using the floral-dip method (Clough and Bent, 1998). T_2_ progeny with a single copy of *BrMYB2* gene introduction and performed with the best purple coloration were selected from independent lines, such as Line 02, Line 06, Line 14, Line 27, Line 32, Line 46, and Line 49; T_3_ homozygous lines were generated from these lines for subsequent experiments (He et al., 2020b). The T_3_ seedlings of these *Arabidopsis* lines were collected at the stage of ca. 60 DAS. All the samples were treated with liquid nitrogen and stored at –80°C in a refrigerator (Sanyo, Osaka, Japan) until further study.

### RNA Extraction, cDNA Synthesis, and Gene Expression Analysis

Total RNA extraction, cDNA synthesis, and the quantitative real-time polymerase chain reaction (qRT-PCR) analysis by the IQ5 optical system (Bio-Rad, United States) was carried out as described by He et al. (2016). Briefly, the reported ABG sequences in *Arabidopsis thaliana* downloaded from the TAIR database^1^ were treated as queries to conduct BLASTP searching in the whole genome of *B. rapa* in BRAD database^2^ (Guo et al., 2014; He et al., 2020a). The gene-specific primers for ABGs in *B. rapa* were those used in a recent report (He et al., 2020a), while the gene-specific primers of corresponding homologous ABGs in *Arabidopsis* were designed in Primer Premier 5.0 software (Premier, Vancouver, BC, Canada), all listed in Supplementary Table S1. All the qRT-PCR data were then normalized using the cycle threshold value corresponding to *BrEF-1*α in *B. rapa* and *AtActin2* in *Arabidopsis*, whose primers were verified by the melting curve analysis for specific amplifications. The relative expression of each target gene was calculated using the 2^–ΔΔ*CT*^ formula (Livak and Schmittgen, 2001), using IQ5 software 2.1 (Bio-Rad, United States). Each sample from pooled tissues was analyzed in three technical replicates and three biological replicates were tested in each sample.

### Determination of Total Anthocyanins

After crushing the fresh samples, approximately 1.0 g from each sample was used for the extraction and determination of total anthocyanin content. This was done using a UV–Vis spectroscopy method as previously described (He et al., 2016), and the obtained data were analyzed following the report from Giusti and Wrolstad, 2001. These results are presented as the mean of three biological replicates.

### Gene Location and Phylogenetic Analysis

Related information about ABG locations and chromosomes was downloaded from the BRAD^2^ and TAIR^1^ databases, and maps for the chromosome distribution of these genes involved in anthocyanin biosynthesis in Chinese cabbage and *Arabidopsis* were constructed in MapChart software 2.3 (Voorrips, 2002). The amino acid sequences of ABGs were employed to perform the phylogenetic analysis using MEGA6. 0 with the neighbor-joining statistical method and 1000 bootstrap values (Koichiro et al., 2013). The code numbers of these proteins were provided in Supplementary Table S1.

### Data Processing and Statistical Analysis

Expression patterns of ABGs that indicated statistically significant changes in the samples were clustered, using a two-way hierarchical clustering methodology, by PermutMatrix software 1.9.3 (Caraux and Pinloche, 2004), for which the Pearson distance and Ward’s method were used in the data aggregation. The Venn diagram of classification of genes was drawn by the Venn web tool^3^. One-way analysis of variance (ANOVA) was implemented in SPSS 13.0 software (Chicago, United States); statistical differences between means were distinguished by the LSD (least significant difference) and Duncan’s multiple range tests. Pearson correlation coefficients were calculated and tested for its two-tailed probability using a bivariate analysis of the data.

## Results

### Characteristics of Anthocyanin Accumulation in Developing Chinese Cabbages

Compared with the color appearance of developing seedlings of the white heading Chinese cabbage 94S17, those of purple heading Chinese cabbage 11S91 featured nearly identical phenotypes except for an extremely light-purple coloration evident in their petioles (Figures 1A–K); however, the male parent 95T2-5 exhibited various degrees of purple coloring, in both petioles and young leaves (Figures 1C–L). Notably, 11S91 began to accumulate anthocyanins during its leaf head formation that following a tissue-specific pattern: the purple coloration of 11S91 mainly appeared in the interior head from the early head-formation to middle head-formation stage, and the total anthocyanin content and color degree declined from interior heading leaves (126.140 and 338.452 mg kg^–1^ at early and middle head-formation stages, respectively) to external heading leaves (44.471 and 8.005 mg kg^–1^ at early and middle head-formation stages, respectively) (Figures 1N–U). Comparatively, the total anthocyanin content and color degree of these purple proportions were far less in 11S91 than the male parent 95T2-5 during whole plant development; the latter significantly accumulated anthocyanins in a continuous increasing manner such that its total anthocyanins reached up to 517.806 mg kg^–1^ (Figures 1S–U). Interestingly, total anthocyanin content of 11S91 was extremely low, at ca. 10 mg kg^–1^ in developing seedlings but increased dramatically, from 126.140 to 338.452 mg kg^–1^, upon entering into the leaf head development period (Figure 1S). By contrast, the white 94S17 did not display purple coloration, failing to accumulate anthocyanins in these tissues under identical conditions (Figure 1). These results indicated that a significant change to the anthocyanin accumulation mechanism occurred between 94S17 and 11S91 during their leaf head development, whereas the different mechanism between 11S91 and its purple trait donor 95T2-5 occurred from 20 DAS developing seedlings. The purple interior heading leaves of 11S91 inherited the characteristic of anthocyanin accumulation from the purple trait donor 95T2-5, but the phenotypes of seedlings were mainly acquired from the female parent 94S17.

### Except for *BrPAL3.1* and *BrC4H4*, Nearly All PMPGs Were Downregulated Significantly During Both Plant Growth and Head Development of Chinese Cabbages

Most PMPGs showed similar expression patterns during seedling growth and head development in the three types of Chinese cabbage. Nearly all *BrPAL*s, *BrC4H*s, and *Br4CL*s were downregulated from the cotyledon stage with different extent of high expression onward, to subsequent development with extremely low expression, and continued to downregulate in heading leaves (Supplementary Figures S1A–H); the prominent time-point of the reduced expression by these genes mainly occurred at the 20 DAS seedling stage (Supplementary Figure S1). By contrast, expression levels of *BrPAL3.1* and *BrC4H4* showed a significant increase in the developing head, and the trend in the expression of *BrPAL3.1* was similar that found for total anthocyanin content of these heading tissues in the two purple lines; however, three lines had similar degrees of *BrC4H4*’s expression in the plant growth (Supplementary Figures S1, S2F,L). The persistently decreasing of *BrPAL2.1* and *BrPAL2.3* in the seedling developing growth, however, showed higher expression levels in developing head tissues of two purple lines, with about 60-fold and 6-fold expression degrees respectively (Supplementary Figures S1, S2A–H). Notably, only *Br4CL2.1* of the *Br4CL*s was highly expressed in the purple lines, showing relatively high expression in the two head-formation periods (Supplementary Figures S1, S2N–U). These results indicated that the initial phenylpropanoid metabolic pathway was a significant biological process and these PMPGs might play important roles in cotyledons for supplying physiological fundaments during Chinese cabbage development; however, only *BrPAL2.1*, *BrPAL2.3*, *BrC4H4*, and *Br4CL2.1* actively functioned in the first phenylpropanoid metabolic pathway during the head development of purple Chinese cabbages with tissue-specific characteristics, whereas *BrPAL3.1* figures prominently in this pathway during the whole development of purple Chinese cabbages.

### Some EBGs’ Expression Was Fluctuated and Increased During the Plant Growth, and Highly Rose During the Head Development of Purple Chinese Cabbages

**FIGURE 3:**
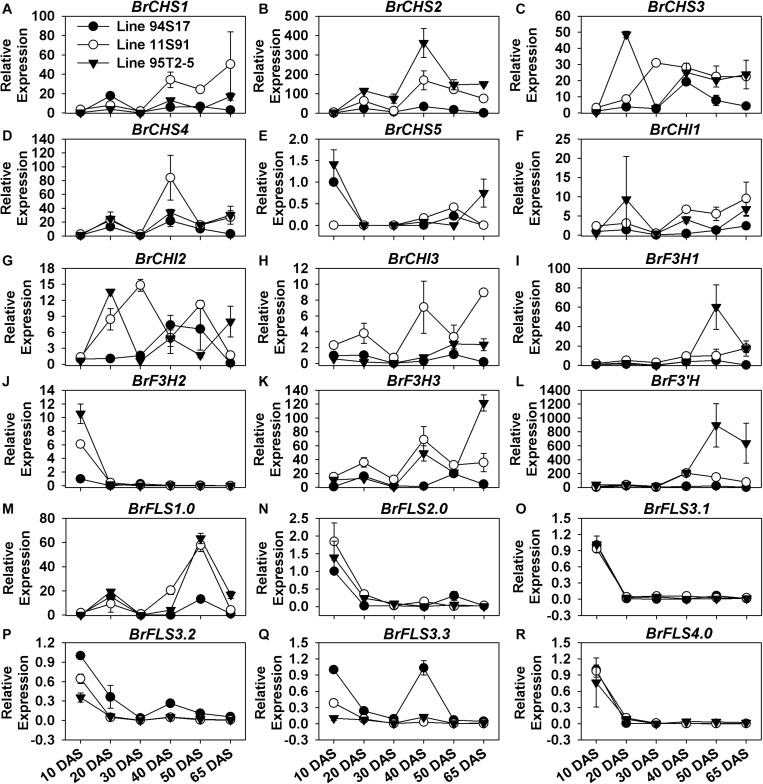
Dynamic gene expression changes of early biosynthesis genes (EBGs) during development of three types of Chinese cabbages. **(A–E)** Expression patterns of *BrCHS*s; **(F–H)** expression patterns of *BrCHI*s; **(I–K)** expression patterns of *BrF3H*s; **(L)** expression patterns of *BrF3’H*; **(M–R)** expression patterns of *BrFLS*s. The 10 DAS seedlings of 94S17 served as the controls in this data analysis, and the 50 DAS and 65 DAS samples were selected from interior head leaves. The values shown are means ± SDs (*n* = 3).

Unlike the PMPGs, the majority of EBGs showed a wave-like pattern of increasing expression during the plants’ growth and head development processes (Figure 3). Their expression levels alternatively increased and decreased from the 10 DAS through 40 DAS seedlings, after which they were moderately downregulated or slightly upregulated in internal heading tissues during head development; these EBGs included *BrCHS1*, *BrCHS2*, *BrCHS3*, *BrCHS4*, *BrCHI1*, *BrCHI2*, *BrCHI3*, *BrF3H3*, and *BrFLS1.0* (Figure 3). For example, they were upregulated in 20 DAS seedlings at first, then downregulated in the 30 DAS stage, upregulated again in 40 DAS seedlings, but sharply downregulated in 50 DAS interior heads, and subsequently upregulated in 65 DAS interior heads (Figure 3). Interestingly, these EBGs were also highly expressed in the head-formation stages, but underwent a decline trend from the internal to external tissues in the two purple Chinese cabbages (Supplementary Figure S3). Certain members, however, such as *BrCHS5*, *BrF3H2*, and the *BrFLS*s (but not *BrFLS1*) involved in flavonol biosynthesis were considerably downregulated during the whole plant development, especially in 20 DAS seedlings (Figure 3 and Supplementary Figure S3). Notably, only *BrF3H1* and *BrF3’H* showed a different expression pattern in the plants’ development, in that they were increasingly upregulated after sowing until the early head-formation process, after their expression decreased slightly in the middle head-formation stage (Figures 3I,L); specially, *BrF3’H* was abnormally and highly expressed with the value up to about 200-fold and 1000-fold in 11S91 and 95T2-5, respectively (Figure 3 and Supplementary Figure S3L). Although the four *BrCHS*s were all highly expressed in developing Chinese cabbages, it was *BrCHS2* which exhibited the highest expression level, whereas *BrCHS1* and *BrCHS4* might have functioned more actively in the middle head-formation stage, and likewise *BrCHS2* and *BrCHS3* at the two head developing periods (Figure 3 and Supplementary Figures S3A–D). Those *BrCHI*s also showed different expression characteristics: *BrCHI1* and *BrCHI3* were expressed more in the two purple lines, whereas *BrCHI2* seemed similarly expressed and functioned between the white and purple lines (Figure 3 and Supplementary Figures S3F–H). In addition, *BrF3H1* showed much higher expression in the early head-formation stage than middle head-formation stage, whereas *BrF3H3* showed an opposite expression pattern in 95T2-5 for these two stages (Supplementary Figures S3I–K). Taken together, we found that the expression characteristics of EBGs were more complicated than PMPGs, and these EBGs showed discontinuous spatiotemporal and tissue-specific expression patterns during the plant growth and head development in Chinese cabbage.

### Key LBGs and Anthocyanin Transport Genes Were Significantly Upregulated During Purple Chinese Cabbages’ Plant Growth and Head Development

**FIGURE 4:**
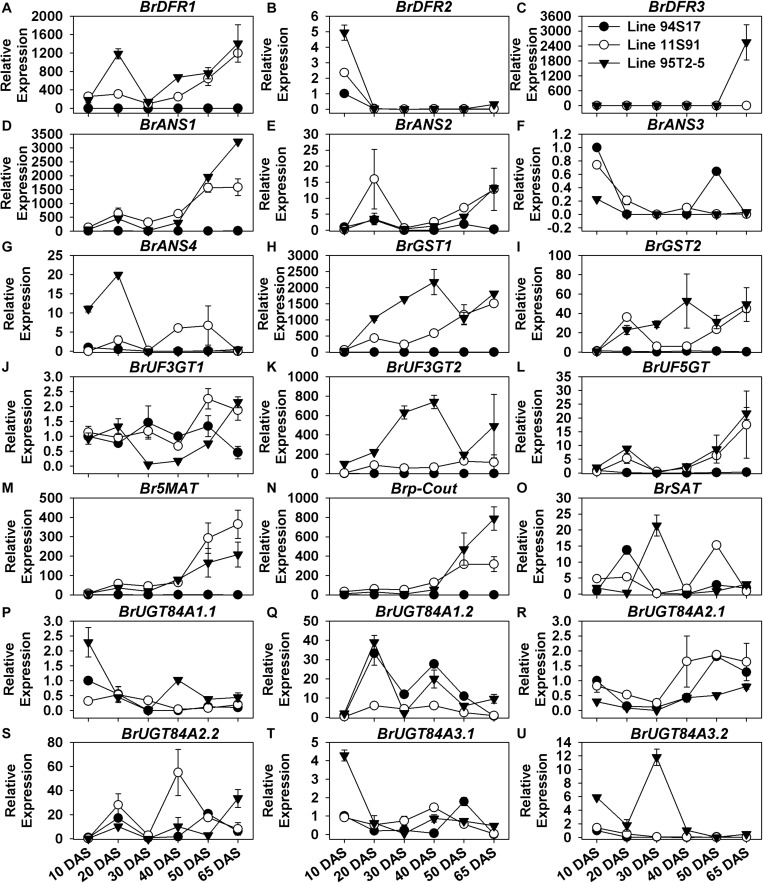
Dynamic gene expression changes of late biosynthesis genes (LBGs) during development of three types of Chinese cabbages. **(A–C)** Expression patterns of *BrDFR*s; **(D–G)** expression patterns of *BrANS*s; **(H,I)** expression patterns of *BrGST*s; **(J–L)** expression patterns of *BrUGT*s; **(M–O)** expression patterns of *BrAT*s; **(P–U)** expression patterns of *BrUGT84A*s. The 10 DAS seedlings of 94S17 served as the controls in this data analysis, and the 50 DAS and 65 DAS samples were selected from interior head leaves. The values shown are means ± SDs (*n* = 3).

The expression of *BrDFR1*, *BrANS1*, *BrANS2*, *BrUF3GT2*, *BrUF5GT*, *BrUF5MAT*, *Brp-Cout*, *BrGST1*, and *BrGST2* was scarcely detected in white 94S17, but their transcription levels were significantly upregulated from seedling growth through the middle head-formation period in both purple lines 11S91 and 95T2-5; in contrast to this pattern, expression levels of *BrDFR2*, *BrANS3*, *BrUGT84A1.1*, *BrUGT84A3.1* kept decreasing in these development stages (Figure 4 and Supplementary Figure S4). Additionally, some LBGs also showed spatial-specific characteristics in Chinese cabbage during its growth: *BrDFR3* was only upregulated in 95T2-5 at the 65 DAS stage, whereas *BrANS4* was highly expressed at about 20-fold in cotyledons and 20 DAS seedlings of 95T2-5 and fivefold in the 40 DAS and 50 DAS tissues of 11S91 (Figures 4C,G); the transcription of *BrUGT84A2.1* only slightly increased from 1-fold to 2-fold in the development of 11S91 and 94S17, whereas *BrUGT84A3.2* was mainly upregulated in 95T2-5 in the 30 DAS stage (Figures 4R,U). Similar to aforementioned EBGs with wave-like expression patterns, the expression levels of sinapic acid: UDP-glucose glucosyltransferase genes *BrUGT84A1.2* and *BrUGT84A2.2* were fluctuated ca. 20-fold, marked by successive upregulation and downregulation during plant growth (Figures 4Q,S). Focusing on the transcription of LBGs and *BrGST*s during the head development, we found that the aforesaid genes *BrDFR1*, *BrANS1*, *BrANS2*, *BrUF3GT2*, *BrUF5GT*, *BrUF5MAT*, *Brp-Cout*, *BrGST1*, and *BrGST2* were all highly and particularly expressed in purple 11S91 and 95T2-5 and also significantly correlated with total anthocyanin content, showing greater hundreds of expression fold changes in internal heading leaves than external leaves; however, *BrUF3GT1* had similar expression levels among three Chinese cabbages (Supplementary Figures S4A–N). However, *BrDFR3* was highly upregulated in 95T2-5 only (Supplementary Figure S4C); *BrUGT84A2.1* only showed augmented expression in the heads of 11S91 and 94S17 (Supplementary Figure S4R); *BrUGT84A3.2* maintained high transcription levels in the 65 DAS outer tissues of 95T2-5 (Supplementary Figure S4U). Taken together, these results further indicated that the key LBGs *BrDFR1*, *BrANS1*, *BrANS2*, *BrUF3GT2*, *BrUF5GT*, *BrUF5MAT*, *Brp-Cout*, as well as the transport genes *BrGST1* and *BrGST2*, were actively participating in the late biosynthesis, modification, and transportation of anthocyanins during purple Chinese cabbages’ development, especially in the purple head growth of 11S91. Nevertheless, some of them were upregulated earlier in 20 DAS seedlings of 95T2-5, namely *BrDFR1*, *BrGST1*, *BrGST2*, and *BrUF3GT2*; several LBGs featured tissue- or spatial- specific characteristics in the growth and development of Chinese cabbages, such as *BrDFR3*, *BrANS4*, and *BrUGT84A3.2*.

### *BrMYB2* Was Tightly Correlated With Anthocyanin Accumulation During Plant Development, Whereas Other Factors Might Selectively Participate in the Process

**FIGURE 5:**
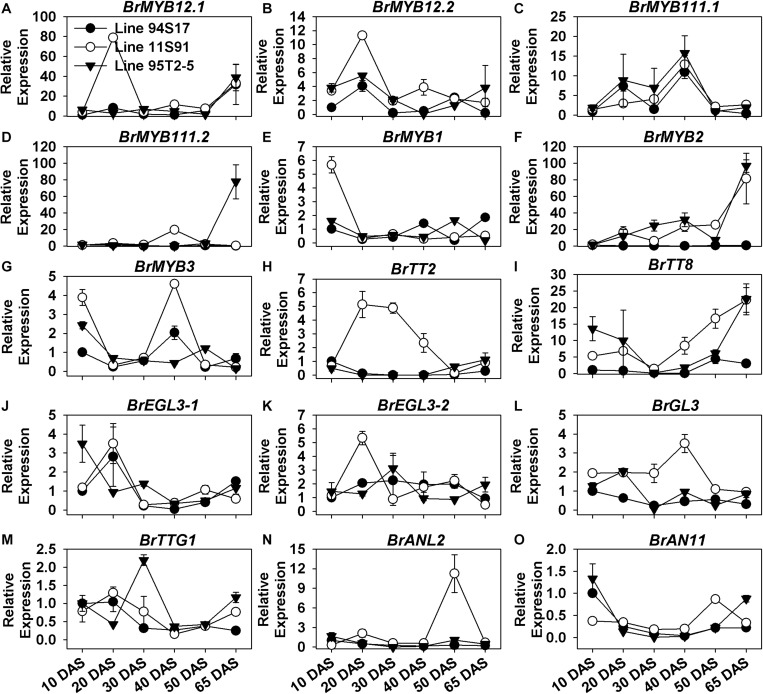
Dynamic gene expression changes of positive regulatory genes during development of three types of Chinese cabbages. **(A–H)** Expression patterns of R2R3-MYB regulatory genes; **(I–L)** expression patterns of basic-helix-loop-helix (bHLH) genes; **(M–O)** expression patterns of *BrTTG1*, *BrANL2*, and *BrAN11*. The 10 DAS seedlings of 94S17 served as the controls in this data analysis, and the 50 DAS and 65 DAS samples were selected from interior head leaves. The values shown are means ± SDs (*n* = 3).

BrMYB12.1 and BrMYB12.2 are highly homologous to AtMYB12, and BrMYB111.1 and BrMYB111.2 are highly homologous to AtMYB111 (Figure 2); meanwhile, AtMYB12 and AtMYB111 share a structural and functional similarity for activating EBGs, such as *AtCHS*, *AtCHI*, *AtF3H*, and *AtFLS1* in *Arabidopsis* (Stracke et al., 2010). However, the expression patterns of *BrMYB12.1*, *BrMYB12.2*, *BrMYB111.1*, and *BrMYB111.2* were differed from each other during the growth and development of Chinese cabbages (Figure 5 and Supplementary Figures S5A–D). For example, both *BrMYB12.1* and *BrMYB12.2* were induced and upregulated in 20 DAS seedlings of 11S91 (Figures 5A,B); however, *BrMYB12.1*’s expression began to increase slightly later at the 65 DAS stage, whereas *BrMYB111.2* only showed a high induction in 65 DAS tissues in 95T2-5 (Figures 5A,D); similar to aforementioned EBGs with their wave-like expression patterns, *BrMYB12.2* and *BrMYB111.1* were also successively upregulated and downregulated during these development processes (Figures 5B,C). During the head development, however, roles of these MYBs might be different. For instance, both *BrMYB12.1* and *BrMYB111.1* were more highly upregulated in the interior proportions of Chinese cabbages, especially in the middle head-formation stage of 11S91; conversely, *BrMYB12.2* and *BrMYB111.2* tended to be expressed more in external head tissues of the three lines, with *BrMYB111.2* especially transcribed highly in 95T2-5 (Supplementary Figures S5A–D).

Concerning the regulatory genes in the formation of the MBW complex, we found that only *BrMYB2* of the MYB genes was upregulated and tightly correlated with anthocyanin accumulation during plant growth, whose expression maintained a slow but increasing tendency during seedling growth, and indicated a severely increase in the head-formation process from 50 DAS interior tissues to 65 DAS internal tissues, in both 11S91 and 95T2-5 (Figure 5F). Comparatively, *BrMYB1* showed decreasing expression during 11S91’s growth, yet *BrMYB3* was highly expressed in cotyledons but also displayed an elevated expression pattern at 40 DAS seedling stage in 11S91 and 50 DAS head stage in 95T2-5, albeit small in magnitude (Figures 5E–H). Regarding the bHLHs, *BrEGL3-1* was expressed the most in 94S17 and 11S91 in the 20 DAS stage, but it was also highly expressed in 95T2-5 in its cotyledon stage; afterward, the gene underwent reduced expression during the rest of the plant development process in the three lines (Figure 5J). Comparatively, the expression of *BrEGL3-2* rose in the 20 DAS stage and 30 DAS stage in 11S91 and 95T2-5, respectively, after which its transcription reduced and remained low at about only onefold during subsequent development (Figure 5K). Notably, the expression of *BrTT8* decreased from cotyledons through young 30 DAS seedlings and then increased to about 25-fold in the head tissues of 11S91 and 95T2-5; however, it was barely expressed in 94S17 (Figure 5I). Together, these results implied that *BrMYB2* played a key role in anthocyanin accumulation, and it might be active and induced for the anthocyanin biosynthesis from the early head-formation stage in 11S91 and its purple trait donor, with the bringing forward of the upregulation point of *BrTT8* to about 40 DAS seedling stage. Although greater expression of *BrGL3* and *BrTTG1* was discernable during young seedlings’ growth, they were downregulated in the old seedlings, with that of *BrTTG1* remaining a slightly elevated tendency during the head development (Figures 5L,M).

In addition, only *BrMYB2* and *BrTT8* were highly correlated with the color and the total anthocyanin content during the head development process, and these two important regulatory genes were highly upregulated in the inner leaves of 11S91 and 95T2-5 (Figure 1 and Supplementary Figure S5). Interestingly, *BrEGL3-1* showed similar expression characters in these purple lines, but it was also highly expressed in the developing head of 94S17 (Supplementary Figure S5J); however, *BrEGL3-2* and *BrTTG1* were expressed more in external heading tissues, together with the other regulatory genes *BrGL3* and *BrEGL3-1*, in 94S17 (Supplementary Figure S5). For the genes *BrMYB1*, *BrMYB3*, *BrTT2*, *BrANL2*, and *BrAN11*, their expression was tissue- and material- specific in the three lines. For example, some of these genes were upregulated in external leaves of 11S91 in its middle head-formation stage, but *BrANL2* and *BrAN11* were upregulated in the interior tissues of 11S91 in the early head-formation stage (Supplementary Figures S5E–O).

**FIGURE 6:**
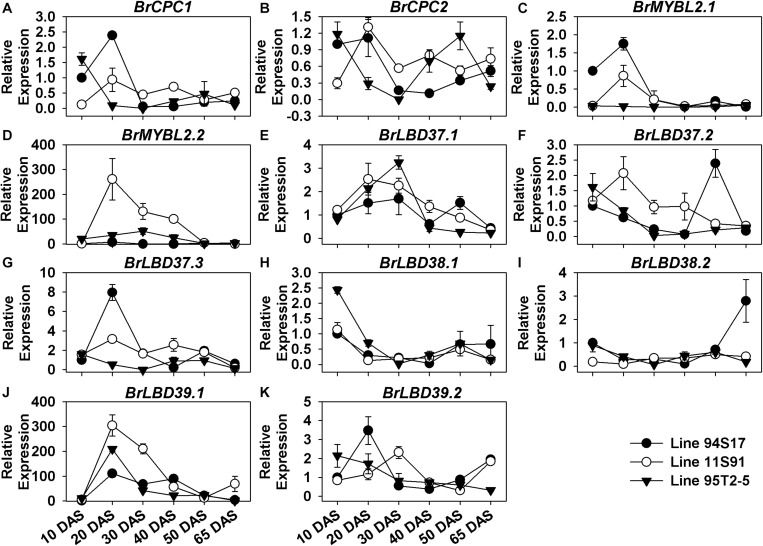
Dynamic gene expression changes of negative regulatory genes during development of three types of Chinese cabbages. **(A–D)** Expression patterns of R3-MYB regulatory genes; **(E–K)** expression patterns of lateral organ boundary domain (LBD) genes. The 10 DAS seedlings of 94S17 served as the controls in this data analysis, and the 50 DAS and 65 DAS samples were selected from interior head leaves. The values shown are means ± SDs (*n* = 3).

Furthermore, the negative regulatory genes also showed special, differential expression patterns during the growth and development of Chinese cabbages. During plant growth, *BrCPC1* and *BrCPC2* were increasingly expressed from cotyledons through 20 DAS seedlings in both 11S91 and 94S17, but their expression levels declined slightly from the 20 DAS stage onward, staying at ca. 0.5-fold until head development; however, *BrCPC1* was scarcely expressed in 95T2-5 but the expression pattern of *BrCPC2* was irregular and differed among three lines (Figure 6 and Supplementary Figures S6A,B). Comparatively, *BrMYBL2.1* was highly expressed in 94S17 in both 20 DAS seedlings and middle formation heads, whereas *BrMYBL2.2* was highly upregulated in the developing seedlings and the none-purple external leaves of 11S91 and 95T2-5 (Figure 6 and Supplementary Figures S6A–D). The majority of LBD genes—namely *BrLBD37.1*, *BrLBD37.3*, *BrLBD38.1*, *BrLBD39.1*, and *BrLBD39.2*—were downregulated after 10 DAS or 20 DAS young seedling stages, and their corresponding high expression peaks mainly appeared in the old plants (Figures 6E–K). During the declining expression of *BrLBD37.2* and *BrLBD38.2* through plant development, two pronounced increases appeared in 94S17, at the 50 DAS and 65 DAS stages, respectively (Figures 6E–K). Importantly, only *BrLBD39.1* was highly upregulated in the growth and development of all three Chinese cabbages, become more active in the 20 DAS seedlings ranging from ca. 100-fold to ca. 300-fold in them but then manyfold diminished in the subsequent head-formation periods (Figure 6J). Results also suggested that *BrLBD37.1*, *BrLBD37.2*, *BrLBD37.3*, *BrLBD38.1*, and *BrLBD39.1* were all more actively in external heading leaves with material-specific features: *BrLBD37.1* and *BrLBD37.2* were expressed at higher levels in 94S17 at the early head-formation stage; *BrLBD37.3* and *BrLBD39.1* were more highly upregulated in the external leaves of both 95T2-5 and 11S91, but at the two head development stages (Supplementary Figures S6E–K). Taken together, these results indicated that the regulatory gene *BrMYB2* plays a leading role in the anthocyanin accumulation of purple heading Chinese cabbage 11S91’s growth and development, whereas *BrTT8* functioned redundantly during its young seedling growth and head development phases; in addition, other positive and negative factors—such as *BrMYB12*s, *BrMYB111*s, *BrMYBL2*s, *BrLBD37.3*, and *BrLBD39.1*—might also participate in the interaction or response network of the anthocyanin regulation in spatiotemporal-specific ways.

### Introduction of *BrMYB2* Activates the Anthocyanin Biosynthesis Pathway Under the Upregulation of PMPGs, EBGs, LBGs, and Transcriptional Genes in *Arabidopsis*

**FIGURE 7:**
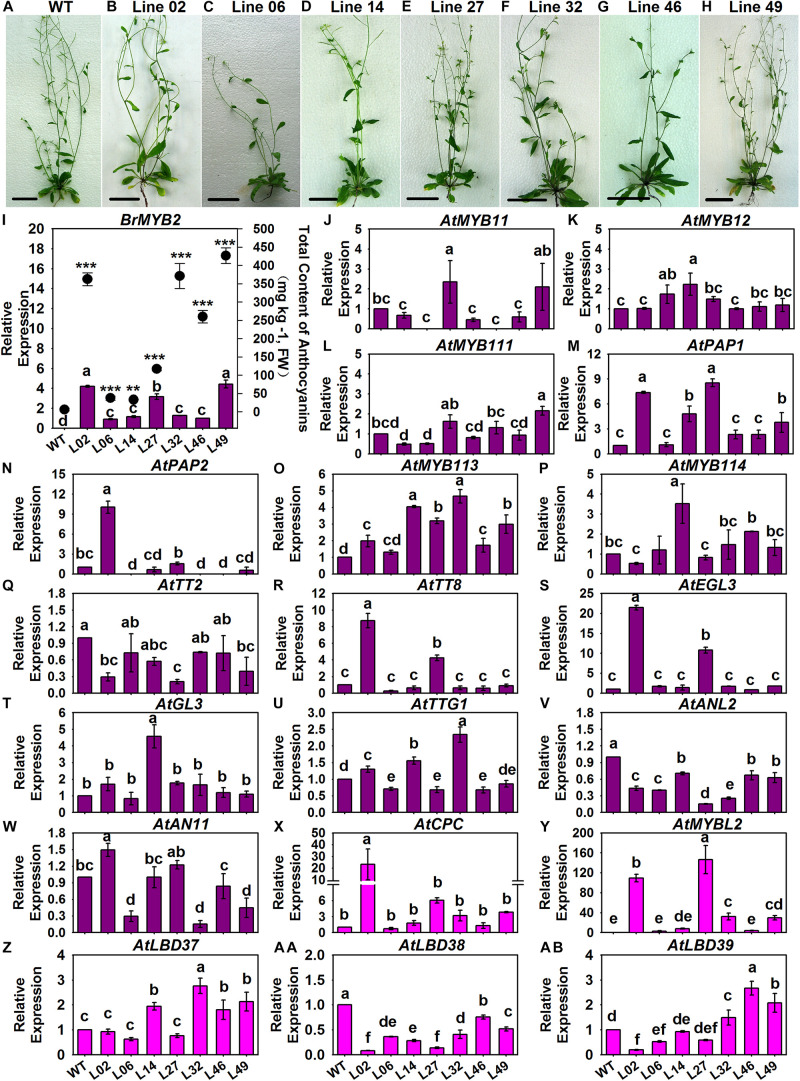
Total anthocyanin content and expression patterns of the regulatory genes in different *BrMYB2-*overexpressing *Arabidopsis* lines. **(A–H)** Appearance of the wild type (WT) *Arabidopsis* and the *BrMYB2*-transgenic lines. **(I)** Total anthocyanin content of the samples and their quantitative real-time PCR (qRT-PCR) analyses of *BrMYB2*, for which Line 46 of the T_3_ *BrMYB2* lines served as the control; the ‘**’ and ‘***’ above each symbol for anthocyanin content indicates highly significant and extremely significant differences, at *p* < 0.01 and *p* < 0.001, respectively. **(J–W)** Expression patterns of positive regulatory genes; **(X–AB)** expression patterns of negative regulatory genes; the WT line served as the control. The values shown are means ± SDs (*n* = 3), and the different letters above each column are significantly different at *p* < 0.05 according to Duncan’s test. The scale bar is 4.5 cm.

In our recent publication addressing the anthocyanin biosynthesis mechanism of heading Chinese cabbage, the R2R3-MYB regulatory gene *BrMYB2* with a short intron 1 isolated from 11S91 combined with map-based cloning and coexpression analysis methods was identified as the key gene governing the accumulation of anthocyanins (He et al., 2020b). Here, we investigated the expression patterns of 54 ABGs in related *BrMYB2* transgenic *Arabidopsis* (namely, Line 02, Line 06, Line 14, Line 27, Line 32, Line 46, and Line 49), including three ABGs without reported orthologs in *B. rapa*, namely *AtUGT84A4*, *AtFLS6*, and *AtMYB11* (Guo et al., 2014); the other 51 ABGs of *Arabidopsis* showed high homology to the aforementioned 86 ABGs of *B. rapa*. The expression degree of *BrMYB2* in aforesaid transgenic lines was 1.0–4.5 times higher than that in Line 46, and these transgenic lines displayed purple coloring in the stems, roots, and rosette leaf stalk bases of 65 DAS seedlings when compared with the wild-type (WT) *Arabidopsis*; however, there was no *BrMYB2* expression detected in WT *Arabidopsis* (Figures 7A–I). Meanwhile, we also measured the total anthocyanin content in these tested transgenic plants. The WT *Arabidopsis* showed a non-purple phenotype in its aboveground parts, with an extremely low anthocyanin content of 5.97 mg Kg^–1^, coupled with negligible *BrMYB2* expression, whereas the transgenic plants had variously high concentrations of anthocyanins, which ranged from 33.045 mg Kg^–1^ in Line 14 up to 426.415 mg Kg^–1^ in Line 49 (Figure 7I). Moreover, Line 49 had the highest anthocyanin content at 426.415 mg Kg^–1^, followed by Line 32, Line 02, Line 46, Line 27, Line 06, and Line 14 (Figure 7I). Yet the ranking of *BrMYB2*’s expression levels differed little from that of total anthocyanin content: that is, Line 49 still maintained the highest expression level of *BrMYB2*, at 4.4285-fold, followed by Line 02, Line 27, Line 32, Line 14, Line 46, and Line 06 (Figure 7I). Accompanied by the high upregulation of *BrMYB2*, we found that crucial R2R3-MYB genes, such as *AtMYB11*, *AtMYB12*, and *AtMYB111*, which mainly target EBGs in *Arabidopsis*, also underwent slight induction in the partial transgenic lines (Figures 7J–AB). In stark contrast, some regulatory genes that participate in the formation of MBW complexes were significantly upregulated in these lines, including the R2R3-MYB genes *AtPAP1*, *AtMYB113*, and *AtMYB114*, the bHLH genes *AtTT8* and *AtEGL3*, and a WD40 gene, *AtTTG1* (Figures 7M–U). Notably, three negative regulatory genes *AtMYBL2*, *AtLBD37*, and *AtLBD39* were also activated in partial *BrMYB2* overexpression lines (Figures 7X–AB).

**FIGURE 8:**
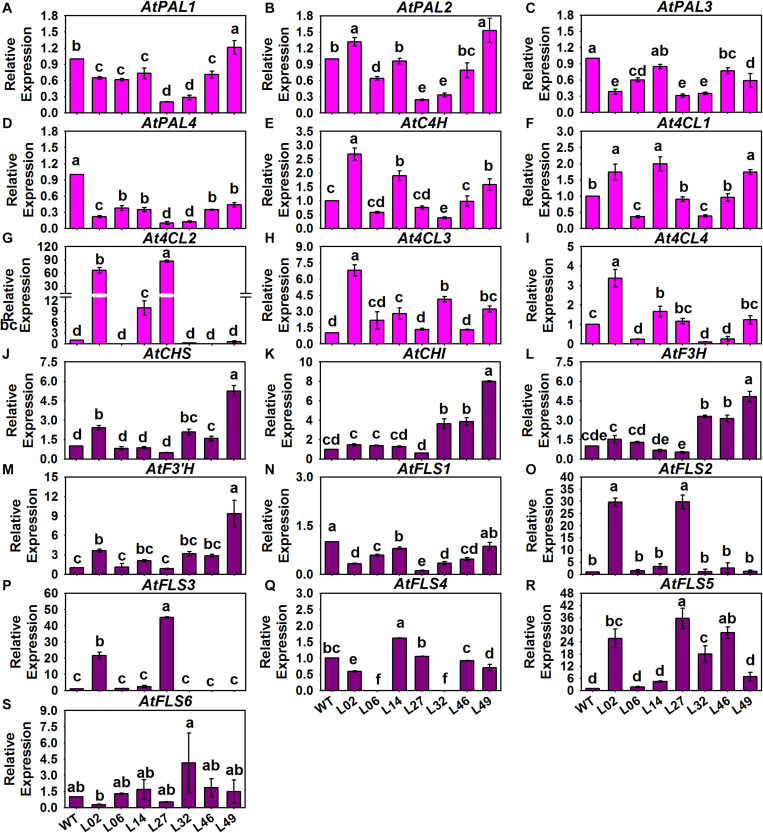
Expression patterns of phenylpropanoid metabolic pathway genes (PMPGs, **A–I**) and EBGs **(J–S)** in different *BrMYB2* overexpression *Arabidopsis* lines. The WT line served as the control, and the values shown are means ± SDs (*n* = 3). The different letters above each column are significantly different at *p* < 0.05 according to Duncan’s test.

**FIGURE 9:**
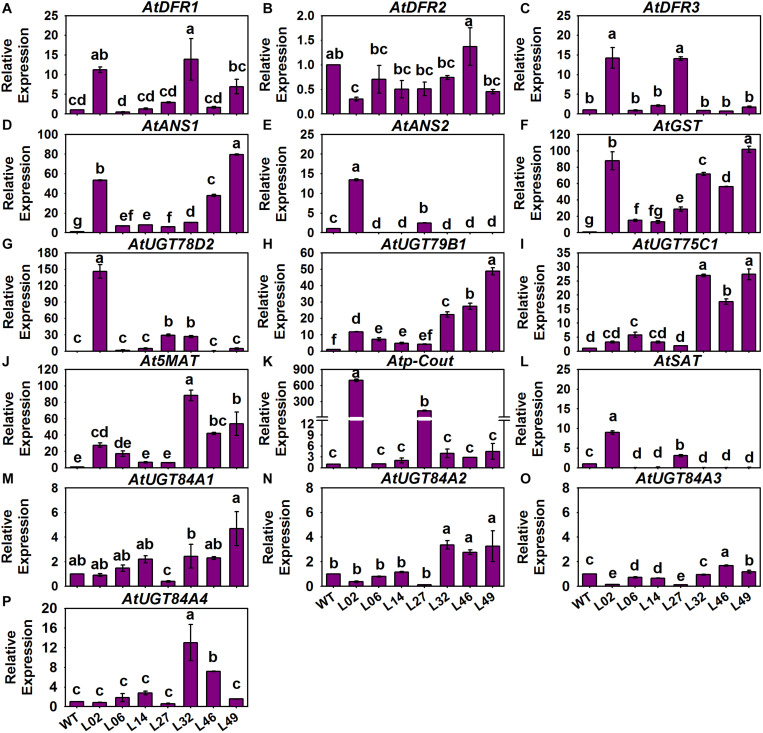
Expression patterns of LBGs and the anthocyanin transport gene in different *BrMYB2*-overexpressing *Arabidopsis* lines. **(A–E)** Expression patterns of LBGs; **(F)** expression patterns of *AtGST*; **(G–P)** expression patterns of LBGs involved in anthocyanins’ modification. The WT line served as the control, and the values shown are means ± SDs (*n* = 3). The different letters above each column are significantly different at *p* < 0.05 according to Duncan’s test.

With regard to the structural genes in these tested *Arabidopsis*, we found that only *AtPAL2* of the *AtPAPL*s underwent a slight 1.5-fold induction in Line 02 and Line 49, and there were no significant differences in expression for the other *AtPAL*s between the control and tested lines (Figures 8A–D). However, *AtC4H* and the *At4CL*s of PMPGs were strongly induced in the transgenic lines, especially the *At4CL2* which exhibited a novel high level of expression in the tested Line 02 and Line 14 (Figures 8E–I). In detecting the EBGs, we found that nearly all of them—including *AtCHS*, *AtCHI*, *AtF3H*, *AtF3’H*, *AtFLS2*, *AtFLS3*, and *AtFLS5*—differed in their respective upregulation among the transgenic lines, but *AtFLS1*, *AtFLS4*, and *AtFLS6* had expression levels indistinguishable from the control (Figures 8J–S). For the LBGs and a transport gene, *AtDFR1*, *AtANS1*, *AtUGT78B1* (*AtUF3GT2*), *AtUGT75C1* (*AtUF5GT*), and *AtGST* were significantly activated in most tested transgenic lines, undergoing fold-changes that ranged from a few to about a hundredfold (Figure 9). Comparatively, some LBGs were only upregulated in partial transgenic lines that had high anthocyanin content, consisted of *AtDFR3*, *AtANS2*, *AtUGT78D2* (*AtUF3GT1*), *At5MAT*, *Atp-Cout*, *AtSAT*, and the all *AtUGT84A*s (Figure 9). Collectively, these results suggested that the exogenous introduction of *BrMYB2* was able to elicit the upregulation of the entire flavonoid biosynthesis pathway in transgenic *Arabidopsis*, and thus not mainly activating the late biosynthesis pathway in Chinese cabbage. Some ABGs might have become operational in most tested plants, whereas the other ABGs were only upregulated in the partial lines. In addition, several members might not respond the upregulation of *BrMYB2*, such as the regulatory genes *AtMYB111*, *AtTT2*, *AtGL3*, *AtANL2*, *AtAN11*, and *AtLBD38*; the PMPGs *AtPAL1*, *AtPAL3*, and *AtPAL4*; the EBGs *AtFLS1* and *AtFLS6*, and the an LBG *AtDFR2*.

## Discussion

Red or purple heading Chinese cabbage not only provides health benefits but also acts as an important germplasm. Recent studies demonstrated that the anthocyanin-rich extracts of red Chinese cabbage are able to reduce the risk of vascular inflammatory diseases and have an anti-inflammatory effect on LPS-stimulated RAW 264.7 cells (Joo et al., 2018; Kwak et al., 2020). The red or purple germplasm of Chinese cabbage has been well discussed when adopting different conceptions of breeding via hybridization (Zhang et al., 2016; Lee et al., 2018; He et al., 2020b). Among them, Zhang et al. (2016) created a purple head Chinese cabbage by crossing a common Chinese cabbage with a red color *B. juncea* using the embryo rescue technique and continuous backcrossing, in which the purple genes were translocated onto chromosome A02; in another breeding case of purple Chinese cabbage, interspecific-crossing between a green Chinese cabbage and a red cabbage was used, combined with recurrent backcrossing to acquire red aneuploid individuals of Chinese cabbage (Lee et al., 2018), for which a series of upregulated ABGs were reported (Rameneni et al., 2020). Unlike those breeding approaches, we relied on the intersubspecific-crossing between a white head Chinese cabbage and a purple flowering Chinese cabbage, with the control of the key *BrMYB2* gene located on chromosome A07 (He et al., 2016, 2020b). Apart from generating new purple heading Chinese cabbages, reports of the purple non-heading Chinese cabbage (2n = AA = 20) have also emerged in recent years. For example, investigations of an F_2_ population created via hybridization of a zicaitai and a common caixin (*B. rapa* L. ssp. *parachinensis*) demonstrated that a positive bHLH gene *BrEGL3.2* and a negative R3-MYB gene *BrMYBL2.1*, located respectively on chromosomes A09 and A07, could be two candidate genes controlling anthocyanin biosynthesis (Guo et al., 2015; Zhang X. et al., 2020). In our hybridization breeding, we found that the purple heading Chinese cabbage 11S91 inherited the key *BrMYB2* gene from its purple parent to control anthocyanin biosynthesis (He et al., 2020b), but the novel phenotype of it incorporated characteristics from both parents during its plant growth processes. Here, we found that the 11S91 was more similar in appearance to the common white parent in the seedlings and outer heading leaves as these developed, whereas it inherited the purple anthocyanin accumulation from the purple donor in the interior heading leaves during its head development (Figure 1). Therefore, it followed that these germplasms harbored different genetic backgrounds to synthesize anthocyanins, despite having similar purple tissues albeit different breeding origins.

Anthocyanins usually accumulate through a gradual process in plants, this being a largely studied subject in horticulture. For example, studies of ornamental crops mainly concentrate on the developing of their flowers and petals: five transcriptional factor genes—*MiMYB1*, *MibHLH1*, *MibHLH2*, *MiWDR1*, and *MiWDR2*—were cloned from *Matthiola incana*, in which MiMYB1 interacted with MibHLH1 and MibHLH2 with their especially increased expression found in petals during floral buds’ development, accompanied by the upregulation of *MiF3’H*, *MiDFR*, *MiANS*, and *Mi3GT* as well as the accumulation of anthocyanins (Nuraini et al., 2020). *Malus hupehensis* has a red-to-white coloration during its flower development and the involved fading mechanism is produced by the higher expression degrees of *PAL*, *CHS*, *CHI*, *DFR*, *FLS*, *ANS*, *UFGT*, *MYB10*, and *MYB12* at early red stages than at later white stages (Han et al., 2020). In other work, flavonol content and *FLS* expression both increased prior to anthocyanin accumulation during floral development, but then decreased once anthocyanins were produced in lisianthus plants, accompanied by the essential upregulation of *CHS*, *CHI*, and *F3H* in both flavonol and anthocyanin biosynthesis throughout floral development and the high transcription of *F3′5′H*, *DFR*, and *ANS* in the late pigmentation stage (Noda et al., 2004). For fruit crops, the focus is not on their flowers but rather focused on the anthocyanin coloration that occur as the fruit develops and ripens (Naing and Kim, 2018). In this respect, MdMYBA, MdMYB1, MdMYB9, MdMYB10, MdMYB110a, and MdMYB11 are all identified R2R3-MYBs that regulate anthocyanin biosynthesis in the peel and flesh of apples: MdMYB1, MdMYB10, and MdMYBA have higher homologies and similar functions for activating LBGs; MdMYB110a is mainly responsible for anthocyanin accumulation of red fleshes in fruits; MdMYB9 and MdMYB11 have a relatively distant relationship to the aforesaid MdMYBs, and the module mechanism of them is to respond to jasmonic acid induction and then activate EBGs such as *MdCHS*, *MdCHI*, and *MdF3H*, as well as several LBGs such as *MdANS*, *MdDFR*, and *MdANR*; both MdMYBL2 and MdMYB6 could inhibit anthocyanin biosynthesis via different mechanisms, whereby the former functions as a repressor interacting with MdbHLH3 to indirectly inhibit the upregulation of *MdMYB10*, *MdbHLH3*, *MdDFR*, and *MdUFGT*, while MdMYB6 could inhibit anthocyanin biosynthesis by regulating *MdANS* and *MdGSTF12* during the coloration of developing fruits (Naing and Kim, 2018; Wang et al., 2019; Xu H. et al., 2020). The expression levels of *F3H*, *UFGT2*, *MYB10*, and *bHLH3* might be critical and coordinated for the anthocyanin synthesis in pear fruits’ development, whereas *GST* and key light-responsive genes, such as *COP1*, *PIF3.1*, and *PIF3.2*, played limited roles in its regulation (Wu et al., 2019). In vegetable crops, studies pay particular attention to either vegetative developing or the fruit development. Work with *Solanaceae* species such as chili pepper showed that *CHS*, *CHI*, *F3H*, *ANS*, and *ANP* are downregulated at some developmental stages of an anthocyanin non-accumulator type, whereas these genes together with *ANS*, *DFR*, and *GST* are developmentally upregulated in plant phenotypes with different contents of anthocyanin accumulation (Aza-González et al., 2013). In addition, reports on *Brassica* crops such as red cabbage have investigated their anthocyanin accumulation and gene expression in response to light and dark conditions during seedlings’ development; more recent work has investigated the ABGs’ expression patterns of purple or red *Brassica* crops in some key stages of the plant development (i.e., seedlings, heads, or both), by using transcriptome sequencing techniques, to effectively reveal the potential mechanism of anthocyanin biosynthesis (Mushtaq et al., 2016; Xie et al., 2016; Zhang et al., 2017; Jeon et al., 2018; Rameneni et al., 2020). Based on these analyses, we find that these studies primarily select a special developing period and a series of typical ABGs involved downstream or upstream of the anthocyanin biosynthesis pathway, the comparisons between the different developing periods, homologous copies, and tissues of plants combining with close genetic backgrounds were rarely studied. Here, to uncover the unique mechanism of anthocyanin biosynthesis, a total of 86 ABGs previously identified in *B. rapa* and linked to various functions involved in phenylpropanoids, lignins, flavonols, and anthocyanins biosynthesis, were analyzed in a purple heading Chinese cabbage and its parents ranging from young seedlings to late head tissues. Meanwhile, the corresponding 51 orthologs and three ABGs (*AtUGT84A4*, *AtMYB11*, and *AtFLS6*, without homologous copies in the reported *B. rapa* genome) of *Arabidopsis* were also investigated in *BrMYB2* overexpression lines to unearth new evidence and insight into anthocyanin biosynthesis.

**FIGURE 10:**
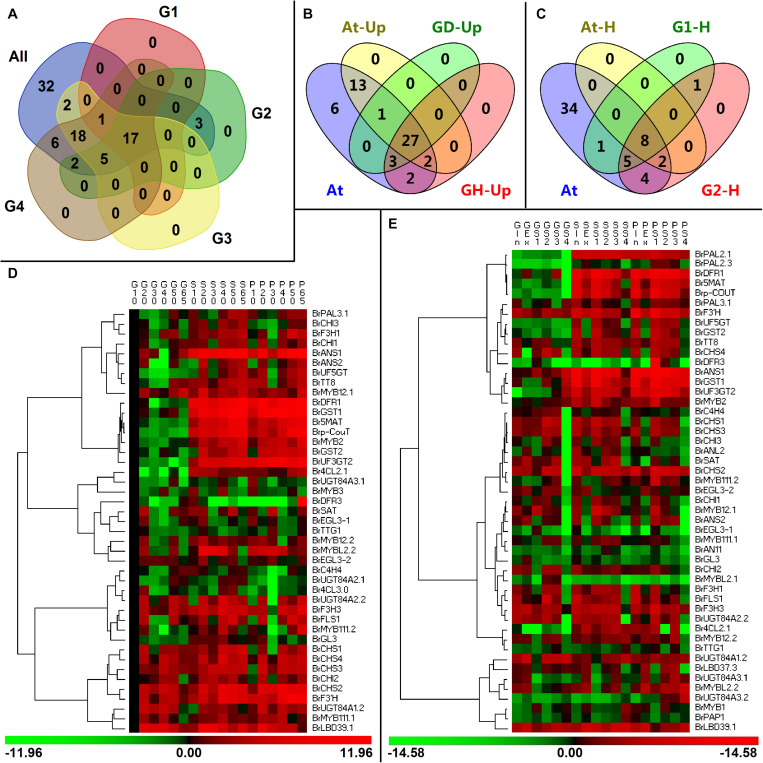
Comparisons of differentially expressed anthocyanin biosynthesis genes (ABGs) in different developing periods of Chinese cabbage and *Arabidopsis*. **(A)** Classification of ABGs in Chinese cabbage. All: the tested 86 ABGs of *Brassica rapa*; G1 and G2 are the classified ABGs which showed significant correlations with total anthocyanin content during plant development and head development, respectively; G3 and G4 show the upregulated ABGs during the plant development and head development, respectively. **(B)** Classification of upregulated ABGs in Chinese cabbage and *Arabidopsis*. At, the tested 54 ABGs in *Arabidopsis*; At-Up, upregulated ABGs in transgenic *Arabidopsis*; GD-Up, upregulated ABGs during Chinese cabbage development; GH-Up, upregulated ABGs during Chinese cabbage head development. **(C)** Classification of ABGs which had significant correlations with total anthocyanin content in Chinese cabbage and *Arabidopsis*; At, the tested 54 ABGs in *Arabidopsis*; At-H, G1-H, and G2-H are classified ABGs showed significant correlations with total anthocyanin content in transgenic *Arabidopsis*, development Chinese cabbage plants, and developing Chinese cabbage heads, respectively. **(D,E)** Hierarchical clustering analyses of expression patterns in upregulated genes during the plant growth **(D)** and head development **(E)** of three Chinese cabbages. The expression data were log2-normalized, clustered using PermutMatrix software, and analyzed with the Pearson distance and Ward’s method. Red boxes indicate upregulation, and green boxes indicate downregulation; the color brightness is directly proportional to the expression ratio. The first capital letters ‘G’, ‘P,’ and ‘S’ are different samples of 94S17, 95T2-5, and 11S91, respectively.

In our exploration, the investigated anthocyanin biosynthesis members clustered in a clade subjected in a family usually showed high homology and might have high functional similarity (Figures 2C–F), but they were expressed with spatiotemporal specificity. Our analysis showed that 17 genes including a PMPG *BrPAL3.1*, EBGs such as *BrF3H1*, *BrF3H3*, and *BrF3’H*, LBGs such as *BrDFR1*, *BrANS1*, *BrANS2*, *BrUF3GT2*, *BrUF5GT*, *Brp-CouT*, and *Br5MAT*, transport genes *BrGST1* and *BrGST2*, and several regulatory genes such as *BrMYB111.2*, *BrMYB2*, and *BrTT8*, were tightly correlated with the anthocyanin accumulation in purple *B. rapa* during its plant growth and head development (Figures 10A,D,E), yet *BrDFR3* was only highly upregulated in the tissues of 95T2-5 during head development (Figure 4 and Supplementary Figure S4). However, the PMPGs *BrPAL2.1* and *BrPAL2.3* were upregulated and only highly correlated with anthocyanin accumulation during the head development; the four EBG genes *BrCHS2*, *BrCHS3*, *BrCHS4*, and *BrCHI1* and a MYB gene, *BrMYB12.1*, all having relatively high expression levels in both plant growth and head development, were strongly correlated with the anthocyanin content in heading tissues; *BrUGT84A3.2* and the regulatory genes *BrMYB1*, *BrANL2*, *BrAN11*, *BrMYBL2.1*, and *BrLBD37.3* underwent tissue-specific upregulation in purple 11S91 or 95T2-5 during head development; *Br4CL2.1* was predominately correlated with anthocyanin accumulation in 95T2-5, but had relatively high expression in both 11S91 and 95T2-5 during head development (Figure 10A). Thus, genetic backgrounds together with multi-copied ABGs determine the complexity and diversify of anthocyanin synthesis mechanism in different types or species of plants.

Another important group, with 18 upregulated ABGs, is composed of the PMPG *BrC4H4*; the EBGs *BrCHS1*, *BrCHI2*, *BrCHI3*, and *BrFLS1*; the modification LBGs *BrUGT84A1.2*, *BrUGT84A2.2*, *BrUGT84A3.1*, and *BrSAT* the regulatory genes such as *BrMYB12.2*, *BrMYB111.1*, *BrMYB3*, *BrGL3*, *BrEGL3-1*, *BrEGL3-2*, *BrTTG1*, *BrMYBL2.2*, and *BrLBD39.1* (Figure 10). They were not significantly correlated with the total anthocyanin content during the growth and head development of cabbage, but they might show high tissue-specific expression characteristics. By contrast, the rest of the ABGs (ca. 40% of them) were either expressed at low levels or not strongly correlated with the anthocyanin content (Figure 10). Interestingly, when *BrMYB2* was introduced into *Arabidopsis*, we found that ca. 80% of ABGs were activated and upregulated in the tested transgenic lines (Figure 10B). The results showed that there are 27 ABGs—comprising the PMPGs *C4H* and *4CL2*, EBGs such as *CHI*, *CHS*, *F3H*, and *F3’H*, LBGs such as *DFR1*, *ANS1*, *DFR3*, *UF3GT2*, *UF5GT*, *5MAT*, *p-CouT*, *UGT84A1*, *UGT84A2*, *UGT84A3*, and *SAT*, the transport gene *GST*, and nine regulatory genes: *MYB12*, *MYB111*, *PAP1 (MYB75)*, *PAP2 (MYB90)*, *TTG1*, *EGL3*, *TT8*, *LBD39*, and *MYBL2*—that were upregulated in both *BrMYB2*-overexpressing *Arabidopsis* and the developing Chinese cabbage plants (Figure 10B). Further correlation analysis showed that only structural genes, such as *CHS*, *CHI*, *F3H*, *F3’H*, *DFR1*, *ANS1*, *5MAT*, *UF3GT2*, and *UF5GT*, and a transport gene *GST* were significantly correlated with the total anthocyanin content in *Arabidopsis*, though the most tested ABGs were found upregulated (Figure 10C). These implied that introducing an exogenous *BrMYB2* gene might directly activate the anthocyanin biosynthesis pathway via the highly upregulated EBGs and LBGs, but the associated regulatory genes might be influenced to differing extents in the various lines. Similarly, overexpression of an R2R3-MYB sweet potato gene, ‘*IbMYB1a*’, in *Arabidopsis* induced the upregulation of the structural genes *4CL*, *CHI*, *F3′H*, *DFR*, *AGT*, *AAT* and *GST*, as well as *AtTT8* (bHLH) and *PAP1*/*AtMYB75* (Choo et al., 2013). Differed to *BrMYB2*, overexpression of the radish *RsPAP2* gene (highly homologous to *AtPAP1* and *AtPAP2*) induced the anthocyanin accumulation in *Arabidopsis*, accompanied by the upregulation or downregulation of several ABGs: *AtC4H*, *AtCHS*, *AtANS*, and *AtTT8* were significantly upregulated in both the roots and leaves of *RsPAP2* overepxression plants; however, *AtDFR* was downregulated; *AtPAL1* and *AtCHI* were specifically upregulated in the roots and leaves, respectively; *AtF3H*, *AtUFGT*, *AtPAP2*, and *AtMYB113*, exhibited upregulated expression in the leaves of the overexpression plants but showed the opposite expression pattern in the roots (Fan et al., 2020).

Anthocyanin biosynthesis genes are generally controlled by various transcriptional factors containing the MYB, bHLH, and WD40 factors, which comprise the most well-known MBW complexes in plants (Pei et al., 2019). Researchers and breeders have usually focused on key regulators of anthocyanin biosynthesis; however, much recent work indicates anthocyanin accumulation is usually affected by the interaction of both positive and negative regulators in plants (Naing and Kim, 2018; Chen et al., 2019). In a prime example reported recently, it is thought that the R2R3-MYB positive regulator SlAN2-like activates the upregulation of an R3-MYB negative regulatory gene *SlMYBATV* and a bHLH positive regulatory gene *SlAN1*, whereas the SlMYBATV competes with SlAN2-like to interact with SlAN1, thereby modulating the accumulation of anthocyanins in tomato (Sun et al., 2020). Another case in point is the monkey flower, which also relies on a reaction–diffusion system to form spots in its petals, whose dynamic regulation of the key machinery involved is achieved by two positive regulators and a negative regulator (Ding et al., 2020). BrMYB75, a member of the R2R3-MYB/bHLH complex, regulated anthocyanin biosynthesis by interacting with BrTT8 in turnip (*B. rapa* subsp. rapa ‘Tsuda’) (Zhang Y. et al., 2020). Similar in our study, *BrMYB2* governs the anthocyanin biosynthesis in purple heading Chinese cabbage 11S91 via substantial upregulation of *BrTT8*; yet several negative regulatory genes are also highly upregulated in Chinese cabbages and *BrMYB2*-overexpressing *Arabidopsis* lines when their anthocyanins began to largely accumulate or dwindle in abundance, genes which included *MYBL2*, *LBD37*, and *LBD39.* Hence, it remains unresolved whether there is a competition-and-interaction module formed by these positive and negative regulators that exists in the purple heading Chinese cabbage. Apart from the high accumulation of anthocyanins, an extra interesting phenotype was also found in the selection of *BrMYB2* transgenic *Arabidopsis* processes: after transformation and the plantation, we found that some of the transgenic lines with high overexpression of *BrMYB2* displayed semi-abnormal reproductive development in T_1_ and T_2_ plants, namely some of siliques were shorter, infertile, and the seed number was decreased; however, we can get seeds from them. At that time, we considered that we didn’t plant and manage well these plants, thus we spent much time planting and managing the T_2_ and T_3_ lines. Then we mainly focused on the creation of useful lines with purple appearances and anthocyanin accumulation to subsequent experiments, and we neglected the abnormal reproductive characters in some lines. For example, pure lines in the Line 02, Line 27, and Line 49 with higher expression of *BrMYB2* were abnormal in fertility: some of their siliques were infertile (Figures 7A–H) and the seed number was also decreased. A recent interesting investigation published by Rahim et al. (2019) found that heterogeneous overexpression of peach *MYB10.1* in tobacco not only regulates the flavonoid biosynthesis (such as anthocyanin and proanthocyanidin) in reproductive parts but also plays a role in other processes such as the vegetative and reproductive development. For example, lines showing a strong phenotype exhibited irregular leaf shape and size and reduced plant height; moreover, flowers had reduced length of anther’s filament, non-dehiscent anthers, reduced pistil length, aborted nectary glands, and impaired capsule development, but the reproductive parts including androecium, gynoecium, and petals were more pigmented that in the WT (Rahim et al., 2019). Thus, whether there is a similar molecular mechanism of formation the sterile siliques in the strong *BrMYB2* overexpression lines remains to be investigated, and this phenomenon might supply evidence when overexpression *MYBs* in related crops to improve their nutritional quality by generating anthocyanins.

**FIGURE 11:**
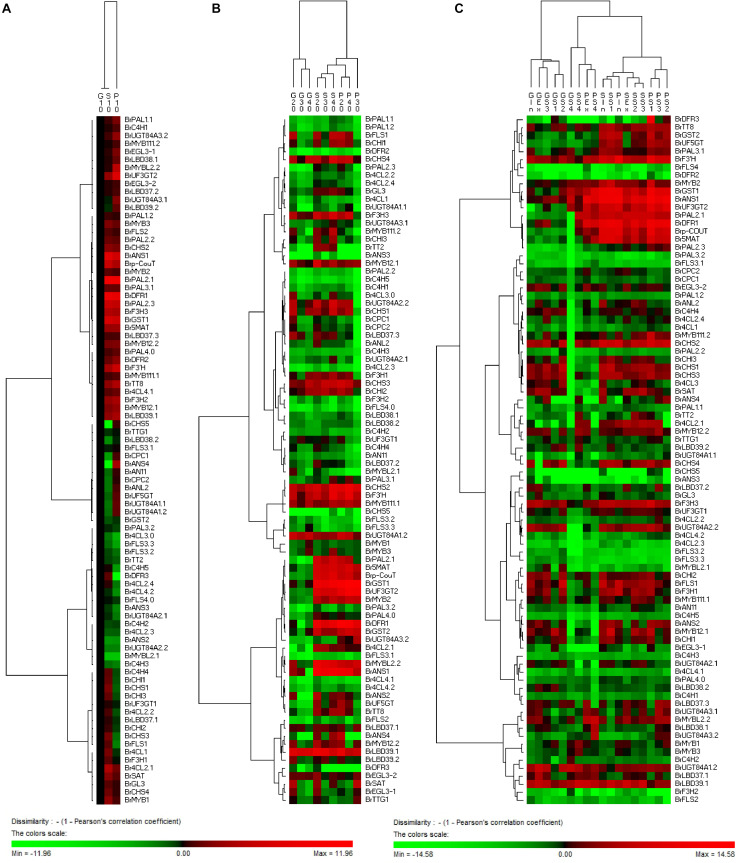
Hierarchical clustering analyses of ABGs’ expression patterns in the cotyledons **(A)**, growing seedlings **(B)**, and developing heading leaves **(C)** of Chinese cabbages. The expression data were log2-normalized, clustered using PermutMatrix software, and analyzed with the Pearson distance and Ward’s method. Red boxes indicate upregulation, and green boxes indicate downregulation; the color brightness is directly proportional to the expression ratio. The first capital letters ‘G’, ‘P,’ and ‘S’ are different samples of 94S17, 95T2-5, and 11S91, respectively.

**FIGURE 12:**
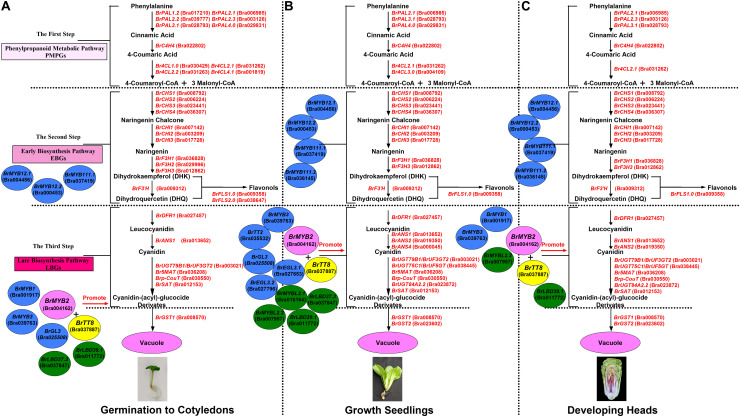
The proposed mode and patterns of anthocyanin biosynthesis in the growth and development of purple heading Chinese cabbage 11S91. **(A)** The cotyledon period, **(B)** the seedling growth stage, and **(C)** the head developing stage.

In addition, based on the clustering analysis of gene expression patterns between 11S91 and its parents, we found that the purple heading Chinese cabbage harbored differential genetic traits vis-à-vis their parents during different stages of growth and development: the cotyledons of 11S91 was classified into a group with 94S17, since it showed similar expression patterns of ABGs to the white parent 94S17, thus implying the cotyledons of the purple heading Chinese cabbage inherited the majority of genetic information from the white parent (Figure 11A); by way of comparison, the growing seedlings and developing head tissues of 11S91 had expression patterns of ABGs that were more similar those of the purple parent (Figures 11B,C); meanwhile, the aforesaid ABGs including PMPGs, EBGs, LBGs, transport genes, positive regulatory genes, and negative regulatory genes were all tightly classified in a group associated with high regulation of *BrMYB2* (Figure 11). These processes indicate that the genes *BrPAL2.1*, *BrPAL3.1*, *BrC4H4*, *Br4CL2.1*, *BrCHS1*, *BrCHS2*, *BrCHS3*, *BrCHS4*, *BrCHI1*, *BrCHI2*, *BrCHI3*, *BrF3H1*, *BrF3H3*, and *BrF’H* may be involved in the early phase of anthocyanin biosynthesis (i.e., the conversion of phenylalanine to dihydroquercetin) in different ontogenic periods of purple Chinese cabbages, whereas *BrFLS1* might compete to enter into the flavonol biosynthesis pathway. The important LBGs *BrDFR1* and *BrANS1*, whose encoded products catalyze the conversion of dihydroquercetin to cyanidin at the late stage of anthocyanin biosynthesis, were both highly upregulated in purple lines at nearly all stages, but not in 94S17 at any stage examined. Meanwhile, high transcript levels of the LBGs *BrDFR1*, *BrANS1*, *BrUF3GT2*, *BrUF5GT*, *Br5MAT*, and *Brp-Cout*, and the final transport of anthocyanins from the cytosol to the vacuole by *BrGST1* and *BrGST2* products, occurred in the purple lines but not in the white line (Figure 12). In the near future, we plan to concentrate our research efforts on unraveling the regulation and interaction of these regulatory factors connected to *BrMYB2* in the purple heading Chinese cabbage.

## Conclusion

Our investigation is the first to systematically demonstrate that the time-point at which purple color formation and the large accumulation of anthocyanins in heading Chinese cabbage 11S91 occurs in the early head-formation stage, whereas the point of the purple trait donor 95T2-5 begins in the young seedling stage. Anthocyanin accumulation in 11S91 is activated by corresponding regulatory and structural genes whose expression levels are co-upregulated during different developmental processes, for which the main LBGs and key regulatory genes were conservative to react, but the PMPGs, EBGs, and some regulatory genes might selectively participate in this pathway (Figure 12). The expression of *BrMYB2* together with *BrTT8*, *BrMYBL2.1*, and *BrMYBL2.2* appears to significantly affect the expression of structural genes related to anthocyanin biosynthesis in purple Chinese cabbages 11S91 and 95T2-5; in particular, the bHLH and R3-MYB factors might harbor specific functions in head color formation. Further, analyzing the molecular mechanism of *BrMYB2* introduced to *Arabidopsis* also showed high similarities regarding the structural genes, with only slight differences with respect to regulatory genes in how the anthocyanin synthesis mechanism operated. This study proposes a special mechanism for purple leaf color formation in developing Chinese cabbage 11S91 and provides a meaningful basis for further research of the purple *Brassicaceae* crops.

## Data Availability Statement

The original contributions presented in the study are included in the article/Supplementary Material, further inquiries can be directed to the corresponding author/s.

## Author Contributions

LZ and QH conceived and designed the experiment and wrote and revised the manuscript. QH, QL, YH, YW, and NZ conducted the RNA extraction, cDNA preparation, qRT-PCR analysis, and material planting. QH and WZ carried out the determination of total anthocyanin content, data processing, statistical analysis, and summary. All the authors have read and agreed to the published version of the manuscript.

## Conflict of Interest

The authors declare that the research was conducted in the absence of any commercial or financial relationships that could be construed as a potential conflict of interest.

## Footnotes

1 https://www.arabidopsis.org/

2 http://brassicadb.org/brad/

3 http://bioinformatics.psb.ugent.be/webtools/Venn/

## Supplementary Material

Supplementary Figure 1 Dynamic gene expression changes of PMPGs during development of three types of Chinese cabbages.

Supplementary Figure 2 Gene expression patterns of PMPGs in the head tissues of different Chinese cabbages.

Supplementary Figure 3 Gene expression patterns of EBGs in the head tissues of different Chinese cabbages.

Supplementary Figure 4 Gene expression patterns of LBGs in the head tissues of different Chinese cabbages.

Supplementary Figure 5 Gene expression patterns of positive regulatory genes in the head tissues of different Chinese cabbages.

Supplementary Figure 6 Expression patterns of negative regulatory genes in the head tissues of different Chinese cabbages.

Supplementary Figure 7 Hierarchical clustering analysis of ABGs’ expression patterns during the growth of Chinese cabbages.

Supplementary Table 1 Primers for the qRT-PCR analysis of genes involved in anthocyanin biosynthesis in *Arabidopsis* and the ABGs in *B. rapa*.

## Abbreviations

4CL: 4-coumarate: CoA ligase
ABGs: anthocyanin biosynthesis genes
ANR: anthocyanin reductase
ANS/LDOX: anthocyanidin synthase/leucoanthocyanidin dioxygenase
AT: acyltransferase
bHLH: basic-helix-loop-helix
Br.: Brassica
C4H: cinnamate 4-hydroxylase
CHI: chalcone-flavanone isomerase
CHS: chalcone synthase
CPC: CAPRICE
DAS: days after sowing
DFR: dihydroflavonol reductase
EBGs: early biosynthesis genes
F3 ′ 5 ′ H: flavanone 3 ′ 5 ′ -hydroxylase
F3H: flavanone 3-hydroxylase
F3’H: flavonoid 3’-hydroxylase
FLS: flavonol synthase
FNS: flavone synthase
GST: glutathione S-transferase
IFS: isoflavone synthase
LBD: lateral organ boundary domain
LBGs: late biosynthesis genes
LAR: leucoanthocyanidin reductase
LSD: least significant difference
MBW: MYB-bHLH-WD40 complex
PAL: phenylalanine ammonia-lyase
PCR: polymerase chain reaction
PMPGs: phenylpropanoid metabolic pathway genes
qRT-PCR: quantitative real-time PCR
UGT: UDP-glucosyltransferase
WT: wild-type.
