# Supplementary material for: Dynamic Changes of the Anthocyanin Biosynthesis Mechanism During the Development of Heading Chinese Cabbage (Brassica rapa L.) and Arabidopsis Under the Control of BrMYB2
Source: Front Plant Sci. 2020 Dec 23;11:593766. doi: 10.3389/fpls.2020.593766 (PMC7785979; doi:10.3389/fpls.2020.593766)
Supplement: Supplementary Figure 1 — Dynamic gene expression changes of PMPGs during development of three types of Chinese cabbages. [file Data_Sheet_1.PDF]

Figure S1. Dynamic gene expression changes of PMPGs during development of three types of Chinese cabbages. (**A–H**) Expression patterns of *BrPAL*s; (**I–M**) expression patterns of *BrC4H*s; (**N–U**) expression patterns of *Br4CL*s. The 10 DAS seedlings of 94S17 were treated as controls in the data analysis, and 50 DAS and 65 DAS samples were selected from interior head leaves. The values are presented as the means ± SDs (n = 3).

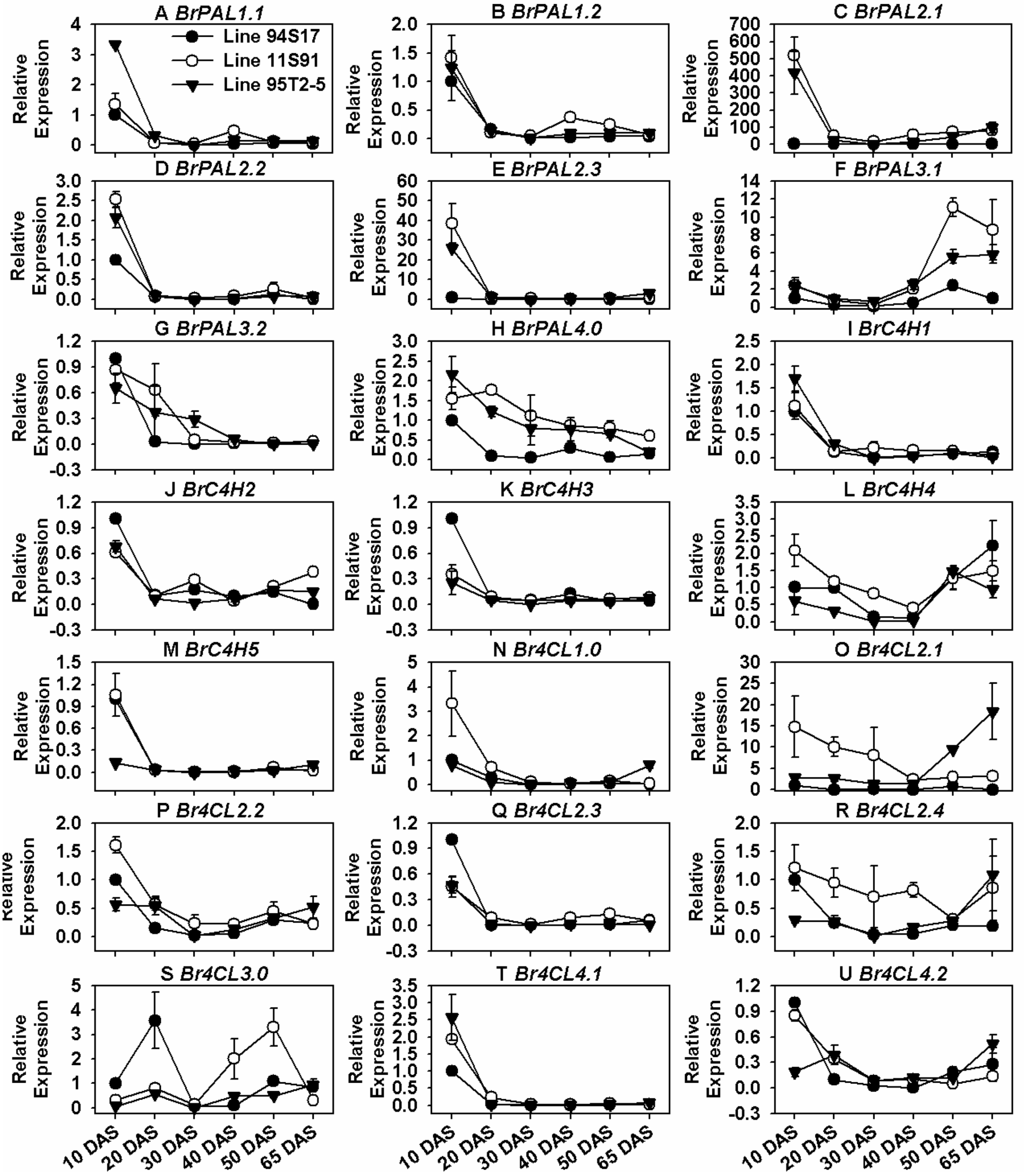

Figure S2. Gene expression patterns of PMPGs in the head tissues of different Chinese cabbages. (**A**–**H**) Expression patterns of *BrPAL*s; (**I**–**M**) expression patterns of *BrC4H*s; (**N**–**U**) expression patterns of *Br4CL*s. The interior purple heading leaves (In) and external leaves (Ex) were collected from 11S91 at the early head-formation stage; the interior heading leaves with deep purple color (S1), the interior heading leaves with light purple color (S2), the exterior heading leaves (S3), and the outer functional leaves (S4) were collected from 11S91 at the middle head-formation stage. The leaf size and positions of 94S17 and 95T2-5 samples at these two stages were collected the same as those of 11S91, and the 10 DAS seedlings of 94S17 were treated as controls in the data analysis. The values are presented as the means ± SDs (*n* = 3).

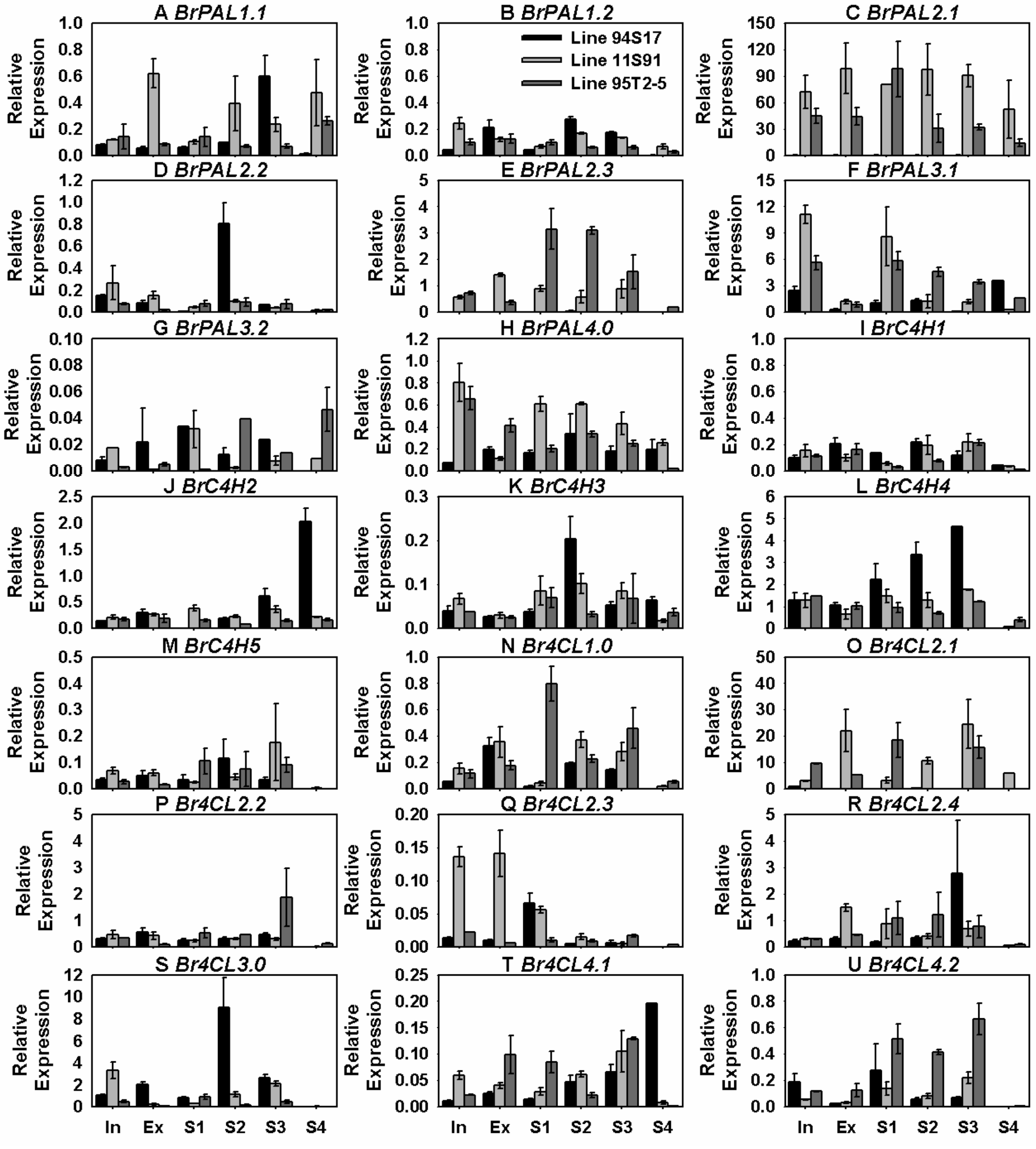

Figure S3. Gene expression patterns of EBGs in the head tissues of different Chinese cabbages. (**A**–**E**) Expression patterns of *BrCHS*s; (**F**–**H**) expression patterns of *BrCHI*s; (**I**–**K**) expression patterns of *BrF3H*s; (**L**) expression patterns of *BrF3'H*; (**M**–**R**) expression patterns of *BrFLS*s. The interior purple heading leaves (In) and external leaves (Ex) were collected from 11S91 at the early head-formation stage; the interior heading leaves with deep purple color (S1), the interior heading leaves with light purple color (S2), the exterior heading leaves (S3), and the outer functional leaves (S4) were collected from 11S91 at the middle head-formation stage. The leaf size and positions of 94S17 and 95T2-5 samples at these two stages were collected the same as those of 11S91, and the 10 DAS seedlings of 94S17 were treated as controls in the data analysis. The values are presented as the means ± SDs ($n = 3$).

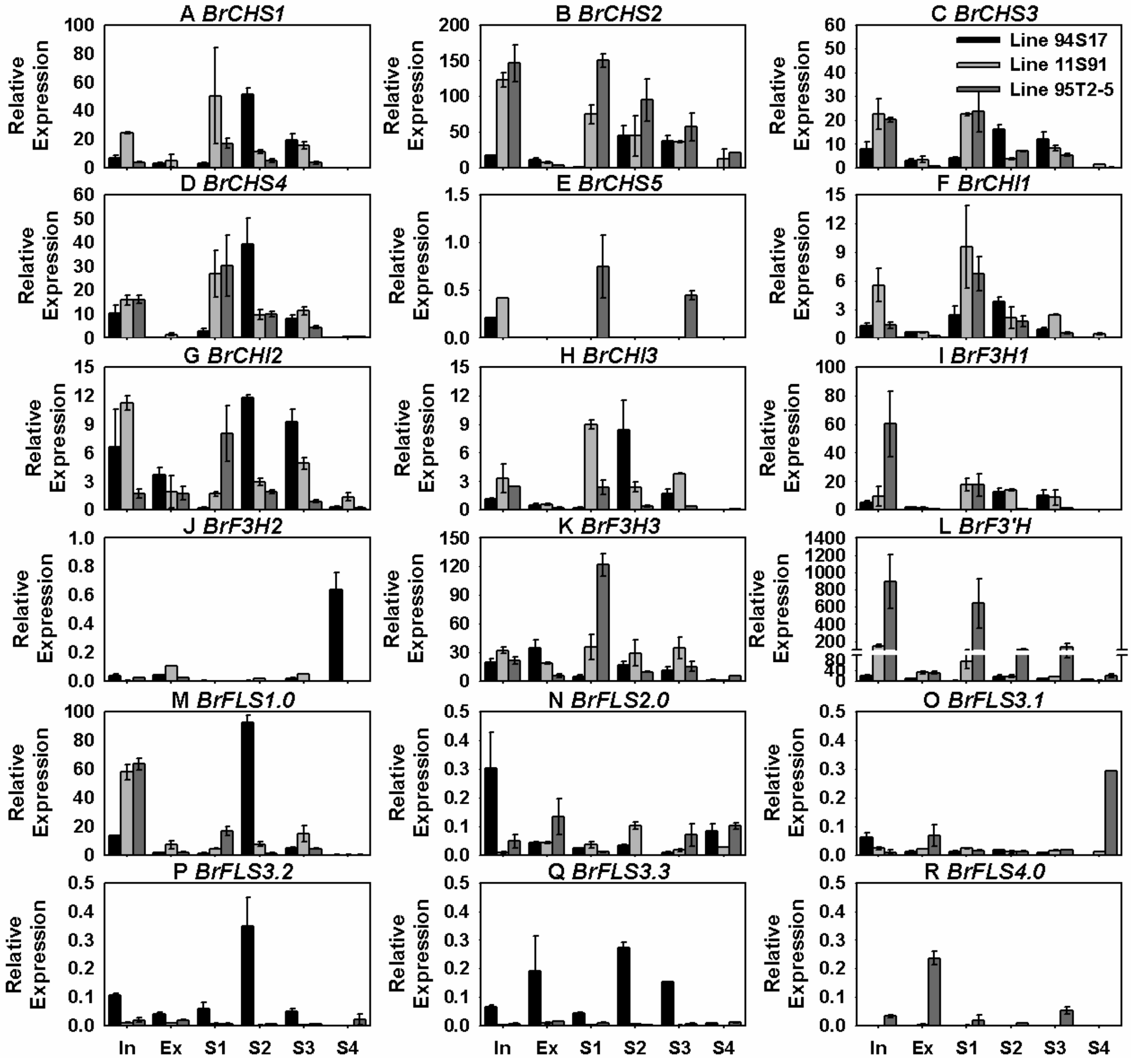

Figure S4. Gene expression patterns of LBGs in the head tissues of different Chinese cabbages. (**A**–**C**) Expression patterns of *BrDFR*s; (**D**–**G**) expression patterns of *BrANS*s; (**H, I**) expression patterns of *BrGST*s; (**J**–**L**) expression patterns of *BrUGT*s; (**M**–**O**) expression patterns of *BrAT*s; (**P**–**U**) expression patterns of *BrUGT84A*s. The interior purple heading leaves (In) and external leaves (Ex) were collected from 11S91 at the early head-formation stage; the interior heading leaves with deep purple color (S1), the interior heading leaves with light purple color (S2), the exterior heading leaves (S3), and the outer functional leaves (S4) were collected from 11S91 at the middle head-formation stage. The leaf size and positions of 94S17 and 95T2-5 samples at these two stages were collected the same as those of 11S91, and the 10 DAS seedlings of 94S17 were treated as controls in the data analysis. The values are presented as the means ± SDs (n = 3).

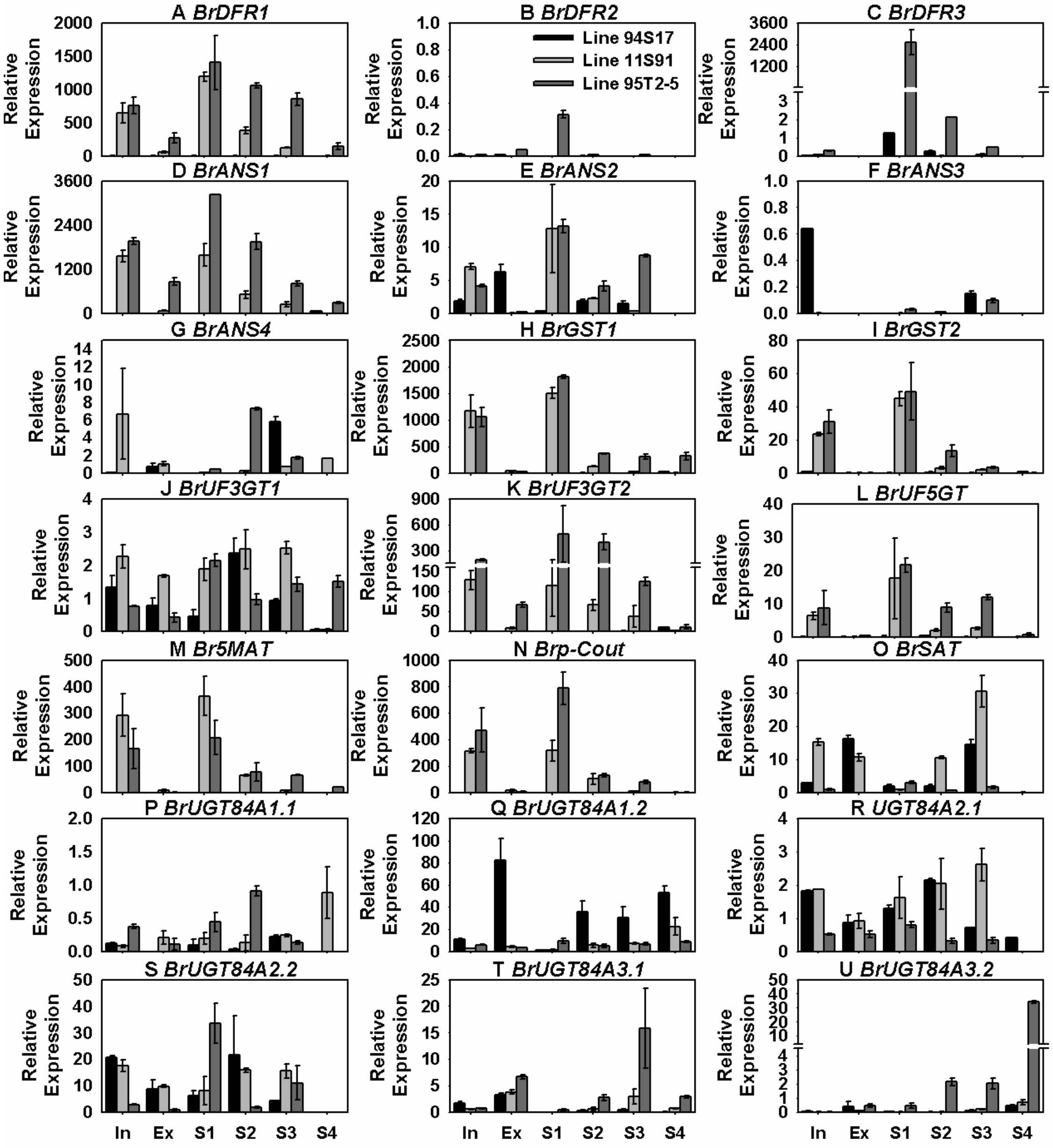

Figure S5. Gene expression patterns of positive regulatory genes in the head tissues of different Chinese cabbages. (**A–H**) Expression patterns of R2R3-MYB genes; (**I–L**) expression patterns of bHLH genes; (**M**) expression patterns of *BrTTG1*. The interior purple heading leaves (In) and external leaves (Ex) were collected from 11S91 at the early head-formation stage; the interior heading leaves with deep purple color (S1), the interior heading leaves with light purple color (S2), the exterior heading leaves (S3), and the outer functional leaves (S4) were collected from 11S91 at the middle head-formation stage. The leaf size and positions of 94S17 and 95T2-5 samples at these two stages were collected the same as those of 11S91, and the 10 DAS seedlings of 94S17 were treated as controls in the data analysis. The values are presented as the means ± SDs (n = 3).

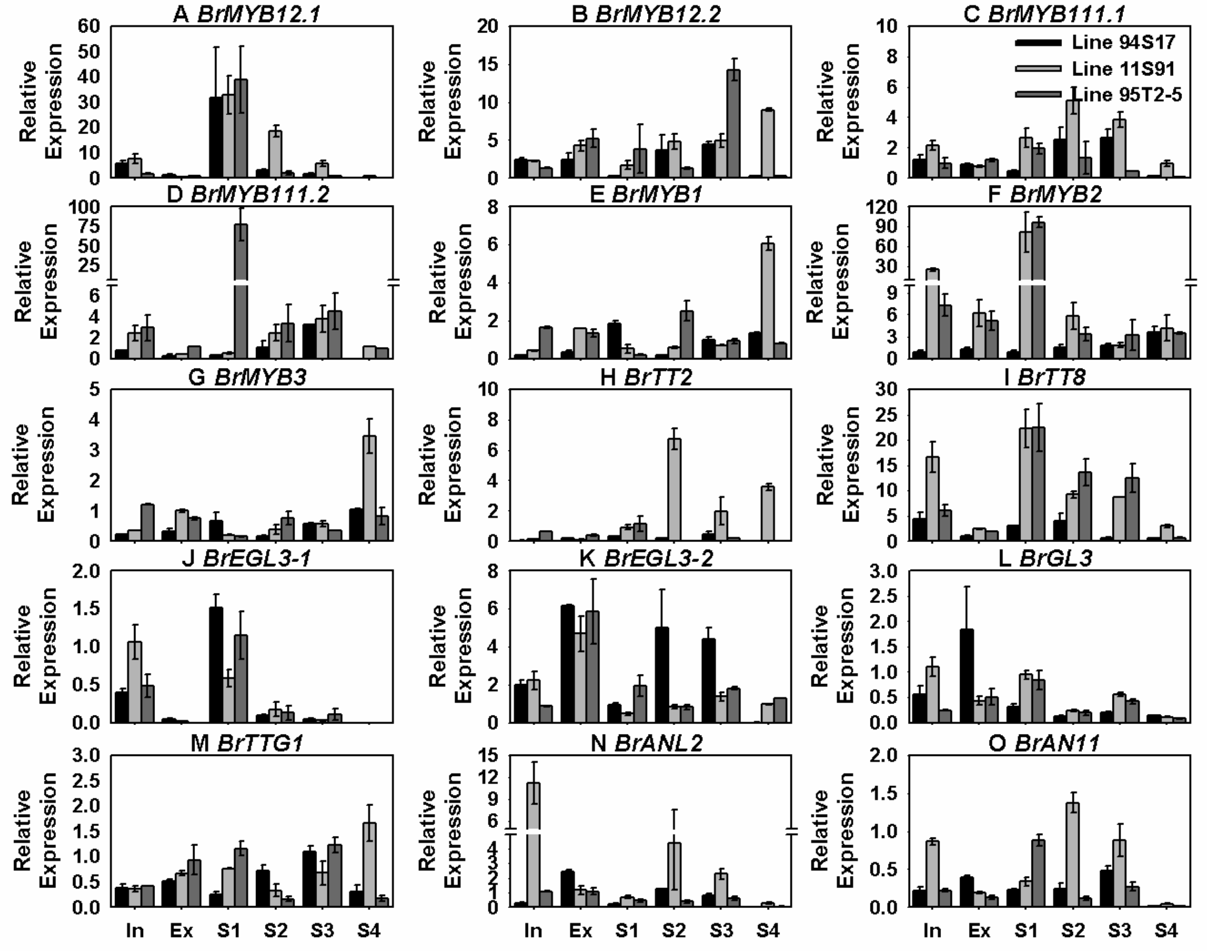

Figure S6. Gene expression patterns of negative regulatory genes in the head tissues of different Chinese cabbages. (**A**–**D**) Expression patterns of R3-MYB genes; (**E**–**K**) expression patterns of LBD genes. The interior purple heading leaves (In) and external leaves (Ex) were collected from 11S91 at the early head-formation stage; the interior heading leaves with deep purple color (S1), the interior heading leaves with light purple color (S2), the exterior heading leaves (S3), and the outer functional leaves (S4) were collected from 11S91 at the middle head-formation stage. The leaf size and positions of 94S17 and 95T2-5 samples at these two stages were collected the same as those of 11S91, and the 10 DAS seedlings of 94S17 were treated as controls in the data analysis. The values are presented as the means ± SDs (*n* = 3).

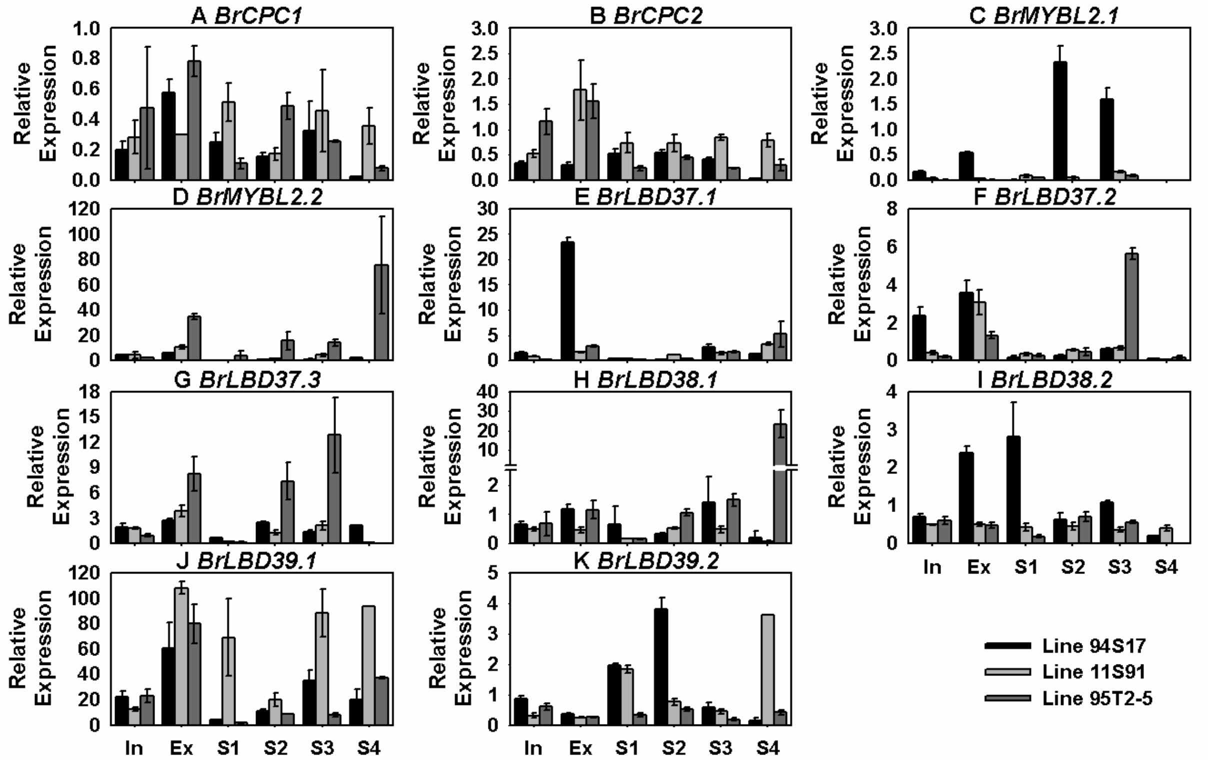

Figure S7. Hierarchical clustering analysis of ABGs' expression patterns during the growth of Chinese cabbages. The expression data were log2- normalized, clustered using PermutMatrix software and analyzed with the Pearson distance and Ward's method. The red boxes indicate upregulation, and the green boxes indicate downregulation; the color brightness is directly proportional to the expression ratio. The first capital letters 'G', 'P,' and 'S' are different samples of 94S17, 95T2-5, and 11S91, respectively.

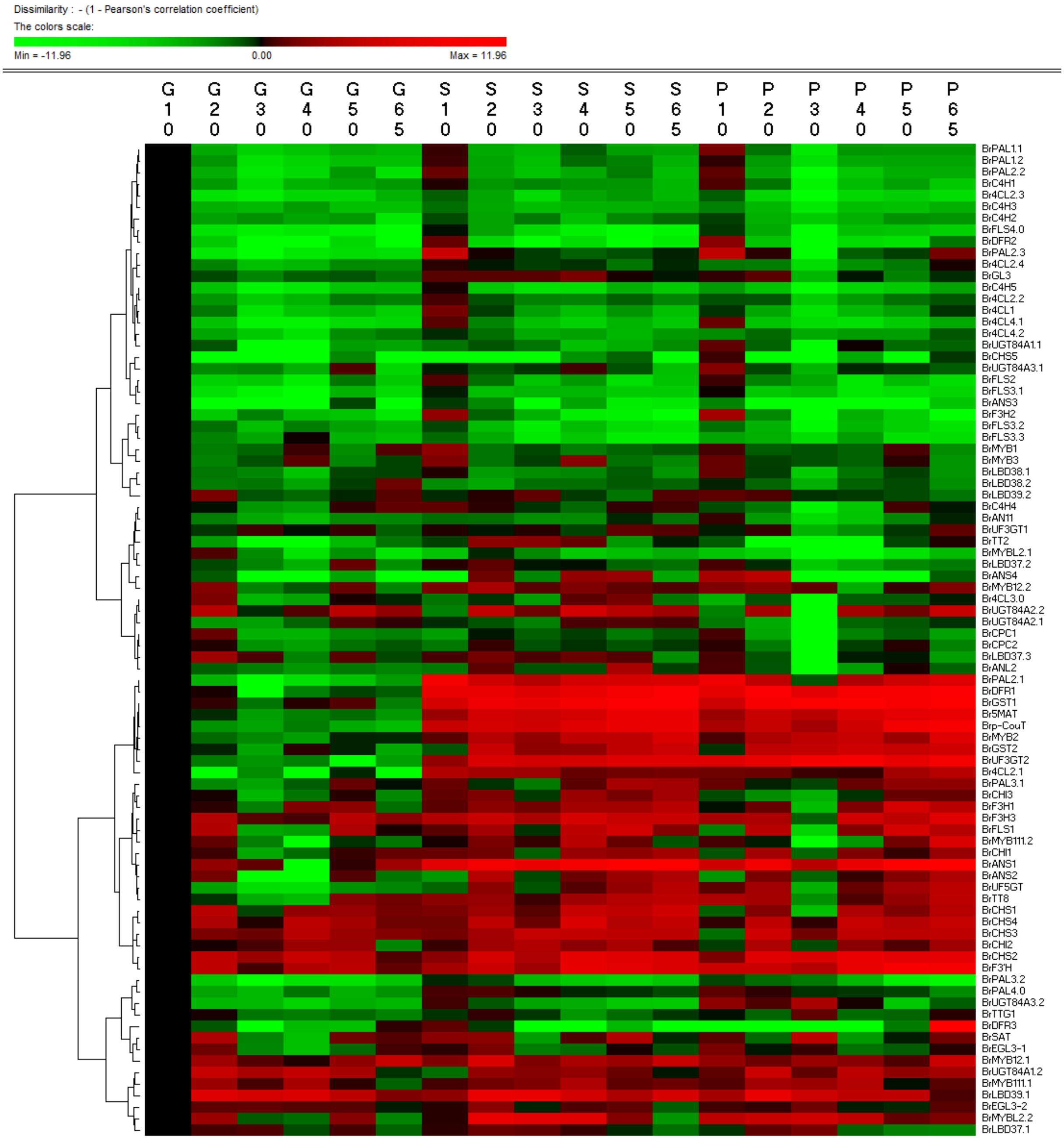

Table S1. Primers for the qRT-PCR analysis of genes involved in anthocyanin biosynthesis in *Arabidopsis* and the ABGs in *B. rapa*. Product length was acquired according to the TAIR database (https://www.arabidopsis.org/). At: *Arabidopsis*; Br: *B. rapa*.

**At Gene**	**At Number**	**Forward Primer (5' to 3')**	**Reverse Primer (5' to 3')**	**Product (bp)**	**Br Gene**	**Br Number**	**Annotation**
AtACT2	AT3G18780	CACTTGCACCAAGCAGCATG	GATTCCTGGACCTGCCTCATC	152	*BrACT2*	Bra037560, Bra022356, Bra001722	ACTIN 2
AtPAL1	AT2G37040	GTGTCGCACTTCAGAAGGAA	GGCTTGTTTCTTTCGTGCTT	72	*BrPAL1.1*	Bra005221	Phenylalanine ammonia-lyase 1
					BrPAL1.2	Bra017210	Phenylalanine ammonia-lyase 1
AtPAL2	AT3G53260	GTGCTACTTCTCACCGGAGA	TATTCCGGCGTTCAAAAATC	77	*BrPAL2.1*	Bra006985	Phenylalanine ammonia-lyase 2
					BrPAL2.2	Bra039777	Phenylalanine ammonia-lyase 2
					BrPAL2.3	Bra003126	Phenylalanine ammonia-lyase 2
AtPAL3	AT5G04230	CAACCAAACGCAACAGCA	CTCCAGGTGGCTCCCTTTTA	78	*BrPAL3.1*	Bra028793	Phenylalanine ammonia-lyase 3
					BrPAL3.2	Bra030322	Phenylalanine ammonia-lyase 3
AtPAL4	AT3G10340	GGTGCACTTCAAAATGAGCT	CAACGTGTGTGACGTGTCC	81	*BrPAL4*	Bra029831	Phenylalanine ammonia-lyase 4
AtC4H	AT2G30490	TGAGTTTGGATCCAGAACGAG	CGTCATGATTCTTCTCATCTTCCT	115	*BrC4H1*	Bra018311	Cinnamate-4-hydroxylase
					BrC4H2	Bra021636	Cinnamate-4-hydroxylase
					BrC4H3	Bra021637	Cinnamate-4-hydroxylase
					BrC4H4	Bra022802	Cinnamate-4-hydroxylase
					BrC4H5	Bra022803	Cinnamate-4-hydroxylase
At4CL1	AT1G51680	TCAACCCGGTGAGATTTGTA	TCGTCATCGATCAATCCAAT	132	*Br4CL1.0*	Bra030429	4-coumarate:CoA ligase 1
At4CL2	AT3G21240	CATCCGTGGCAACCAAATC	GCGACGACAGCAACATCA	240	*Br4CL2.1*	Bra031262	4-coumarate:CoA ligase 2

					Br4CL2.2	Bra031263	4-coumarate:CoA ligase 2
					Br4CL2.3	Bra031265	4-coumarate:CoA ligase 2
					Br4CL2.4	Bra031266	4-coumarate:CoA ligase 2
At4CL3	AT1G65060	GTCGTCTCCGATGATGTGC	TGTTTCGCCGTAGGTGTAGC	189	*Br4CL3*	Bra004109	4-coumarate:CoA ligase 3
At4CL4	AT3G21230	GAATCCGTTCTACACTCAGCC	CCGTTGTCACCGTCATCGT	153	*Br4CL4.1*	Bra001819	4-coumarate:CoA ligase 5
					Br4CL4.2	Bra001820	4-coumarate:CoA ligase 5
AtCHS	AT5G13930	CGCATCACCAACAGTGAACAC	TCCTCCGTCAGATGCATGTG	101	*BrCHS1*	Bra008792	Chalcone synthase
					BrCHS2	Bra006224	Chalcone synthase
					BrCHS3	Bra023441	Chalcone synthase
					BrCHS4	Bra036307	Chalcone synthase
					BrCHS5	Bra020688	Chalcone synthase
AtCHI	AT3G55120	CCGGTTCATCGATCCTCTTC	ATCCCGGTTTCAGGGATACTATC	88	*BrCHI1*	Bra007142	Chalcone isomerase
					BrCHI2	Bra003209	Chalcone isomerase
					BrCHI3	Bra017728	Chalcone isomerase
AtF3H	AT3G51240	CAGATCGTTGAGGCTTGTGAGA	ACGAGTCATATCCGCCACTAAGT	87	*BrF3H1*	Bra036828	Flavanone 3-hydroxylase
					BrF3H2	Bra029996	Flavanone 3-hydroxylase
					BrF3H3	Bra012862	Flavanone 3-hydroxylase
AtF3'H	AT5G07990	GCTCTCGCCGGAGTATTCAA	CCAGCGACGCCTTGTAAATC	74	*BrF3'H*	Bra009312	Flavonoid 3'-hydroxylase
AtFLS1	AT5G08640	CCGTCGTCGATCTAAGCGAT	CGTCGGAATCCCGTGGT	107	*BrFLS1*	Bra009358	Flavonol synthase 1
AtFLS2	AT5G63580	GGTTACACGACGAATCTCAAG	ATCCTCATTACATACTCGGTC	263	*BrFLS2*	Bra038647	Flavonol synthase 2
AtFLS3	AT5G63590	GGATCGACGCGGAGTATAC	GAAATTTGTAGTCTTTGTAGAC	249	*BrFLS3.1*	Bra038648	Flavonol synthase 3

AtFLS4	AT5G63595	TGAAGATGAGCAACGGAAGG	GGTGAGTTCGGGAAATGGC	133	*BrFLS3.2*	Bra029211	Flavonol synthase 4
AtFLS5	AT5G63600	TATCTAGGAGGTATAAACAATTG	TAAGCACATACTCTGCCGTG	241	*BrFLS3.3*	Bra029212	Flavonol synthase 5
					BrFLS4	Bra037747	Flavonol synthase 5
AtFLS6	AT5G43935	TCTCCACCGTGTCTCCTAACC	CCACTCTTCGCTCGCTTTC	102	——	——	——
AtDFR1	AT5G42800	AACGGATGTGACGGTGTTTT	TCCATTCACTGTCGGCTTTA	93	*BrDFR1*	Bra027457	Dihydroflavonol-4-reductase
AtDFR2	AT4G27250	ATTCCTTACTCCATTTGTCCCATC	GCAATCGAACCCATTCTTTTGT	114	*BrDFR2*	Bra019062	Dihydroflavonol-4-reductase
AtDFR3	AT4G35420	CGATGTCCTCGGATTACTGAA	GGGATAGGGAGTGATGGGTAA	210	*BrDFR3*	Bra010535	Dihydroflavonol-4-reductase
AtANS1	AT4G22880	CGATGAAAAGATCCGTGAGA	GCCAATTTACTTCCATAGCCT	215	*BrANS1*	Bra013652	Leucoanthocyanidin dioxygenase
					BrANS2	Bra019350	Leucoanthocyanidin dioxygenase
AtANS2	AT2G38240	CTTGGGGTTGTGAAAGATGC	ATTTTGGGTAGAAGTTTGTCCTC	273	*BrANS3*	Bra017132	Leucoanthocyanidin dioxygenase
					BrANS4	Bra000045	Leucoanthocyanidin dioxygenase
AtANL2	AT4G00730	CTGTATGATTTCTTGCGGAACG	GACACCCTGATCTTGACCTTTG	111	*BrANL2*	Bra037355	ANTHOCYANINLESS 2
AtAN11	AT1G12910	ATCCGATTCAAGATGGTTCC	GGCTAGTGATGGCGAGACGA	132	*BrAN11*	Bra026947	ANTHOCYANIN11
AtUGT78D2（*AtUF3GT1*）	AT5G17050	CACCGCACAATCCAACTCT	GCATTTATCTCCGTCGCCAT	273	*BrUGT78D2(BrUF3GT1)*	Bra023594	Anthocyanidin 3-O-glucosyltransferase
AtUGT79B1（*AtUF3GT2*）	AT5G54060	CAACTGGTTTTCCGTTTCTGGTT	GCTTCCTCGACGGTTGATACAC	64	*BrUGT79B1(BrUF3GT2)*	Bra003021	Anthocyanin 3-O-glucoside: 2-O-xylosyltransferase
AtUGT75C1（*At5GT*）	AT4G14090	CGAAGGCATTACCGTCAGC	GCATCGTGTTCCAAAGCAG	112	*BrUGT75C1(BrUF5GT)*	Bra038445	Anthocyanin 5-O-glucosyltransferase
At5MAT	AT3G	AGCCACGCTCCTCCA	ACGGCATCTTTGTC	102	*Br5MAT*	Bra036208	Malonyl-CoA:anthocyanidin

	29590	CTATC	GTCAGG				5-O-glucoside-6"-O-malonyltransferase
Atp-CouT	AT1G03940	CAGAGCCACTTTTACATTGAGC	TCATCCTTGTCTTCCTCGTTG	183	*Brp-CouT*	Bra030550	HXXXD-type acyl-transferase family protein
AtUGT84A1	AT4G15480	GTAAAGGGATGATTGTGGATTG	CAAAGATTCCATTGTCGAGTTC	107	*BrUGT84A1.1*	Bra039547	Sinapic acid:UDP-glucose glucosyltransferase
					BrUGT84A1.2	Bra012784	Sinapic acid:UDP-glucose glucosyltransferase
AtUGT84A2	AT3G21560	TTCGGGACCGTTGCTTACT	AAAACAAACCGTCGGGACTC	282	*BrUGT84A2.1*	Bra031290	Sinapic acid:UDP-glucose glucosyltransferase
					BrUGT84A2.2	Bra023872	Sinapic acid:UDP-glucose glucosyltransferase
AtUGT84A3	AT4G15490	ATCTCGTTAAGAGATATAACAAGGAGCC	CGTTGTTTATGAGACACGTCACC	51	*BrUGT84A3.1*	Bra039545	Sinapic acid:UDP-glucose glucosyltransferase
					BrUGT84A3.2	Bra039544	Sinapic acid:UDP-glucose glucosyltransferase
AtUGT84A4	AT4G15500	TCGGGCTAGGTTTTCTCCG	TGTAGACAAATCCATCCTCGAAGA	51	——	——	——
AtSAT	At2g23000	CTGCTGCTATAGTCAAGTCTCTTCC	GAGAGAAGAACATCCAGGTCCTC	188	*BrSAT*	Bra012153	Sinapoyl-Glc:anthocyanin acyltransferase
AtGST	AT5G17220	TGGTCGAGGATCTCAAAGTG	TGAATTCTTCACCAGCCAAA	93	*BrTT19.1(BrGST1)*	Bra008570	Transparent Testa 19
					BrTT19.2(BrGST2)	Bra023602	Transparent Testa 19
AtMYB11	AT3G62610	GGCGATTGTAACCCAAGCATT	TCACATGAGGACACGTGGACA	116	——	——	——
AtMYB12	AT2G47460	TGATGGGGAGTTGCATAACATA	AACGACTCCACCGATGGAC	114	*BrMYB12.1*	Bra004456	MYB Domain Protein 12
					BrMYB12.2	Bra000453	MYB Domain Protein 12
AtMYB111	AT5G	AATAACAAGACCAAG	AGAAACATTGTGAG	92	*BrMYB111.1*	Bra037419	MYB Domain Protein 111

	49330	AAGAAGAAGAA	GCCGTC				
					BrMYB111.2	Bra036145	MYB Domain Protein 111
AtPAP1(AtMYB75)	AT1G 56650	AAATGGCACCAAGTT CCTGT	TCAGAGCTAAGTTT TCCTCTCTTGAT	113	*BrMYB1*	Bra001917	Production of Anthocyanin Pigment 1
					BrMYB2	Bra004162	Production of Anthocyanin Pigment 1
					BrPAP1 (*BrMYB3*)	Bra039763	Production of Anthocyanin Pigment 1
AtPAP2(AtMYB90)	AT1G 66390	CAAGAGAGGAAGACT TAGCAATGA	CTCAGATGGGTGTT CCAGTAATT	144	*BrMYB2*	Bra004162	Production of Anthocyanin Pigment 2
					BrMYB1	Bra001917	Production of Anthocyanin Pigment 2
					BrPAP1 (BrMYB3)	Bra039763	Production of Anthocyanin Pigment 2
AtMYB113	AT1G 66370	ATCTTGTTCTTCGCCT TCATAAA	GCATCGTTCATCGT GCTTCTTA	134	*BrMYB1*	Bra001917	Production of Anthocyanin Pigment 1
					BrMYB2	Bra004162	Production of Anthocyanin Pigment 1
					BrPAP1 (*BrMYB3*)	Bra039763	Production of Anthocyanin Pigment 1
AtMYB114	AT1G 66380	GTCTCTTGAGGCAGT GTATTGGT	TTTTCCTGCACCGAT TTAGC	87	*BrMYB2*	Bra004162	Production of Anthocyanin Pigment 2
					BrMYB1	Bra001917	Production of Anthocyanin Pigment 2
					BrPAP1 (BrMYB3)	Bra039763	Production of Anthocyanin Pigment 2
AtTT2	AT5G 35550	TTGATGGTTTGGACT GTGGA	TGAAGTCTCGGAGC CAATCT	153	*BrTT2*	Bra035532	Transparent Testa 2
AtTT8	AT4G 09820	TGAATCAACCCATAC GTTAGACA	GGGGTGTGACATGA GAAGTGT	102	*BrTT8*	Bra037887	Transparent Testa 8
AtGL3	AT5G 41315	AGTGTTTAGCCGTTCT CTTCTAGC	TGTCTTCCGTAATAT GTTCTGTGG	113	*BrGL3*	Bra025508	Glabrous 3
AtEGL3	AT1G 63650	TTGGCACGACCGAAC ATA	TTGATAGTCTGATC TTGTCGATATTGT	100	*BrEGL3.2*	Bra027796	Enhancer of Glabrous 3

					BrEGL3.1	Bra027653	Enhancer of Glabrous 3
AtTTG1	AT5G 24520	TCCTCGAAGATTACA ACAACCG	CGGGAGAGGCTTAA CGGTCAT	72	*BrTTG1*	Bra009770	Transparent Testa Glabrous 1
AtMYBL2	AT1G 71030	AAAACCCGCCTTCGT GC	CCCGCTATCAATGA CCATCT	179	*BrMYBL2.1*	Bra016164	MYB-Like 2
					BrMYBL2.2	Bra007957	MYB-Like 2
AtCPC	AT2G 46410	GGAGACAGAGCAAA GCCAAGG	CCAACGAGTTTATA CATCCGAGAA	118	*BrCPC1*	Bra004539	CAPRICE
					BrCPC2	Bra039283	CAPRICE
AtLBD37	AT5G 67420	TGCTTTGTTTCAGTCG TTGCTC	TGCTCCGTTAACTG GATTGACA	64	*BrLBD37.1*	Bra012164	LOB Domain-containing Protein 37
					BrLBD37.2	Bra031833	LOB Domain-containing Protein 37
					BrLBD37.3	Bra037847	LOB Domain-containing Protein 37
AtLBD38	AT3G 49940	TGCCCTGCTTTGTTTC AGTCTT	CGTTCACCGGATTC ACAGTTCT	64	*BrLBD38.1*	Bra036040	LOB Domain-containing Protein 38
					BrLBD38.2	Bra012913	LOB Domain-containing Protein 38
AtLBD39	AT4G 37540	GAACTCCAACGTCCT GCTTTGT	ATACCAACCGCTCC GTTAACC	86	*BrLBD39.1*	Bra011772	LOB Domain-containing Protein 39
					BrLBD39.2	Bra017831	LOB Domain-containing Protein 39
